# Conformational changes in protein kinase A along its activation cycle are rooted in the folding energetics of cyclic-nucleotide binding domains

**DOI:** 10.1016/j.jbc.2023.104790

**Published:** 2023-05-06

**Authors:** Amy K. Chau, Katherine Bracken, Lihui Bai, Dominic Pham, Lydia L. Good, Rodrigo A. Maillard

**Affiliations:** Department of Chemistry, Georgetown University, Washington, District of Columbia, USA

**Keywords:** protein kinase A (PKA), cyclic AMP (cAMP), conformational changes, allostery, optical tweezers, computational modeling

## Abstract

Cyclic-nucleotide binding (CNB) domains are structurally and evolutionarily conserved signaling modules that regulate proteins with diverse folds and functions. Despite a wealth of structural information, the mechanisms by which CNB domains couple cyclic-nucleotide binding to conformational changes involved in signal transduction remain unknown. Here we combined single-molecule and computational approaches to investigate the conformation and folding energetics of the two CNB domains of the regulatory subunit of protein kinase A (PKA). We found that the CNB domains exhibit different conformational and folding signatures in the apo state, when bound to cAMP, or when bound to the PKA catalytic subunit, underscoring their ability to adapt to different binding partners. Moreover, we show while the two CNB domains have near-identical structures, their thermodynamic coupling signatures are divergent, leading to distinct cAMP responses and differential mutational effects. Specifically, we demonstrate mutation W260A exerts local and allosteric effects that impact multiple steps of the PKA activation cycle. Taken together, these results highlight the complex interplay between folding energetics, conformational dynamics, and thermodynamic signatures that underlies structurally conserved signaling modules in response to ligand binding and mutational effects.

The cyclic-nucleotide binding (CNB) domain is a signaling module found in all kingdoms of life (1). Its evolutionarily conserved structure comprises of a β-subdomain harboring the phosphate-binding cassette for cyclic nucleotide docking, and a α-helical subdomain that contains a N-terminus helical bundle called N3A motif (Fig. 1*A*, inset). Despite high-resolution structures of CNB domains have been available for decades, there remains a lack of understanding in how CNB domains enable the CNB signal to regulate a diverse array of proteins, such as kinases, guanine nucleotide-exchange factors, nucleotide-gated channels, and transcription factors. Moreover, given that proteins containing CNB domains are multimeric, it is also challenging to dissect how the CNB activities are coordinated during cyclic nucleotide signaling.

**Figure 1:**
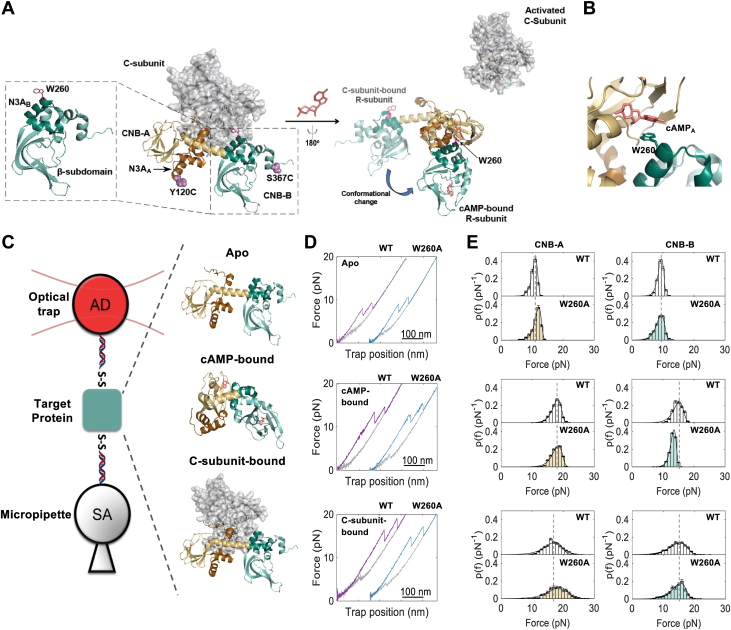
**Functional states of PKA R-subunit probed with optical tweezers.***A*, structure of the inactive PKA holoenzyme (*left*, PDB: 2QCS) and after cAMP binding, the active dissociated regulatory (R) (PDB: 1RGS) and catalytic (C) subunits (*right*). The N3A motifs of the CNB domains are highlighted in *brown* and *dark green*, and the β-sandwiches are highlighted in *gold* and *light green*. The C-subunit-bound and cAMP-bound R subunits were aligned at the CNB-A domain, where the conformational change in the R-subunit from C-subunit bound to cAMP-bound is shown with a *dark green curved arrow*. The R-subunit structure on the *right* is flipped 180 degrees for better visualization. *B*, interactions between W260 and cAMP in the CNB-A domain. *C*, schematic representation of a single-trap optical tweezers assay. Target protein is covalently attached by dsDNA handles modified with either biotin or digoxigenin. The modified ends interact with streptavidin (SA) or anti-dig (AD)-coated polystyrene beads, respectively, to generate a single tether that is held by a micropipette and an optical trap. In this study, the target protein can be in apo, bound to cAMP, or bound to the C-subunit. *D*, force-extension curves of the unfolding of WT (*purple*) and W260A mutant (*blue*) in apo, cAMP-bound, and C-subunit-bound states. Refolding event is shown in *grey*. *E*, unfolding force probability distribution of CNB-A and CNB-B domains in apo (*top*), cAMP-bound (*middle*), and C-Subunit-bound (*bottom*) states. WT is colorless and W260A mutant is in *gold* (CNB-A) or *light green* (CNB-B). The *solid lines* are fit to Equation 1. AD, anti-dig; CNB, Cyclic-nucleotide binding; SA, streptavidin; WT, wildtype.

Across many CNB domain-containing proteins, the regulatory (R) subunit of protein kinase A (PKA) contains two CNB domains in tandem (denoted as CNB-A and CNB-B) connected by a helix termed B/C-helix (Fig. 1*A*). The crystal structures of the R-subunit bound to cAMP (active state) or bound to the PKA catalytic (C) subunit (inactive state) reveal noticeable conformational differences (2, 3). cAMP binding to the CNB domains switches the protein from an elongated to a compact, globular conformation. This conformational change is distinctive at the B/C-helix where it bends at L233 and Y244, bringing the CNB-B domain closer to the CNB-A domain (4). Moreover, this domain motion allows W260 in the CNB-B domain to interact and cap the cAMP docked on the CNB-A domain binding pocket (Fig. 1*B*). Before reaching the final cAMP-bound conformation, the N3A motif in the cAMP-bound CNB-A domain (denoted as N3A_A_ motif) moves aways from the CNB-A β-subdomain and establishes contacts with the B/C helix and CNB-B domain (2, 5).

The change in conformation that the R-subunit experiences makes it an ideal candidate to dissect the molecular mechanisms by which the CNB domains adapt to different functional states along the activation cycle of PKA. In this study, we combined single molecule optical tweezers experiments and computational approaches based on ensemble modeling (6) to probe the conformational dynamics and folding energetics of the two CNB domains as truncated domains or together as part of the R-subunit. By comparing the CNB domains as isolated structures or as part of the R-subunit, we aim to dissect the emerging allosteric properties of these signaling domains as they interact with each other. Moreover, these studies were conducted in the apo, cAMP-bound, and C-subunit-bound states using wildtype and the mutant W260A, a residue located in the CNB-B domain that interacts with the cAMP in the CNB-A domain (Fig. 1*B*), which disrupts interdomain interactions (4, 7). Therefore, this study allows us to dissect the contributions of each CNB domain to cAMP-mediated signaling and to quantitate mutational effects in different functional states.

Our study shows that the CNB domains exhibit unique folding energy landscape signatures in each of the three functional states, underscoring their plasticity to adapt to different binding partners. The mutation W260A exerts differential effects depending on the functional state. For instance, W260A has no effects on the C-subunit-bound state, moderate effects on the stability of the CNB domains in the apo state, and large effects on the stability and dynamics of the CNB domains in the cAMP-bound state. We find that these mutational effects in the cAMP-bound R-subunit are both local and allosteric: (1) Intra-domain interactions between the N3A_B_ motif and its β-subdomain. (2) Disruption of surface contacts between the two CNB domains. (3) Stabilization of the N3A_A_ motif in the distant CNB-A domain. Computational work reveals that the two CNB domains have unique thermodynamic signatures, resulting is different residue connectivity networks despite sharing the same structural makeup. The computational results support the experimental observations of the differential responses of the CNB domains with different binding partners (cAMP or C-subunit). W260A abolishes many of the residue connectivity networks throughout the R-subunit, providing a thermodynamic foundation for the expansive mutational effects of W260A seen experimentally. Altogether, this study underscores the complex interplay between folding energetics, conformational dynamics, and thermodynamic signatures underlying structurally conserved signaling modules in response to ligand binding and mutational effects.

## Results

### Mechanical unfolding trajectories of R-subunit

We used optical tweezers to compare the folding properties of the wild-type and W260A R-subunits. In this experiment, we tethered a single R-subunit between two polystyrene beads using two 370-bp DNA handles covalently linked to the protein *via* disulfide bond linkages at positions flanking the two CNB domains (Y120C and S376C) (Fig. 1*C*, Experimental procedures). By moving the bead in the optical trap away or towards the bead on the micropipette (force-ramp), we monitored in real-time changes in force and extension due to the mechanical unfolding of the tethered protein (Fig. 1*D*). These molecular trajectories revealed two major sudden changes in extension, or rips, that were assigned to unfolding of each CNB domain of the R-subunit (Fig. S1 and Supporting information). In this study, we investigate the folding energy landscape of the R-subunit and the effect of W260A in three functional states: apo, bound to cAMP, or in complex with the C-subunit.

### W260A exerts differential effects depending on the PKA functional state

We first compared the unfolding force distributions of each CNB domain in the wild-type and W260A R-subunits in the apo state. We find that the average unfolding force (F_ave_), for the CNB-B domain was slightly lower than the CNB-A for both the wildtype and the mutant R-subunits (Fig. 1*E* and Table S1). Interestingly, the CNB-A domain in the mutant R-subunit had a statistically higher F_ave_ than that of the wildtype protein, 10.5 ± 1.0 pN for the mutant and 11.3 ± 1.4 pN for wildtype, indicating long-range mutational effects (Table S1, Two-sample Kolmogorov-Smirnov test, *p* ≈ 0). To determine which kinetic parameter accounts for the difference in F_ave_, we extracted the folded state lifetime at zero force (τ_0_) and the distance to the transition state (Δx^‡^) of both CNB domains using the Bell model (Experimental procedures, Equations (1), (2)). The analysis revealed that the mutant CNB-B domain displayed a 24-fold lower τ_0_ relative to the wildtype, whereas the CNB-A domains had similar τ_0_, (Table S1). However, the mutation decreased Δx^‡^ of both CNB-A and CNB-B domains by 15 to 35%. Thus, the mutation W260A in the CNB-B domain exerts both local effects and long-range allosteric effects over the neighboring CNB-A domain.

Given the effect of W260A on both CNB domains in the apo state, we investigated whether these mutational effects are amplified when the protein is bound to cAMP. When the R-subunit is bound to cAMP, the CNB domains of both wildtype and mutant unfolded at higher forces than in the apo state (Fig. 1*D*, middle). Due to interdomain interactions triggered by cAMP binding (Fig. 1*A*, right), the wildtype CNB-B domain unfolded at a force higher than that of the truncation, 14.8 ± 1.5 pN and 12.6 ± 0.9 pN, respectively (Tables S1 and S2) (5). Interestingly, the F_ave_ of the CNB-B domain in the mutant R-subunit was lower by 1.7 pN compared to wildtype, 13.1 pN ± 1.0 pN. This value is indistinguishable from the F_ave_ of the truncated CNB-B mutant domain bound to cAMP, 12.6 ± 0.9 pN (Table S2). In fact, we found that τ_0_ and Δx^‡^ were the same between the W260A CNB-B domain in the R-subunit or as a truncated domain. These results indicate that the CNB domains in the W260A R-subunit unfold independently of each other in the presence of cAMP. Therefore, interdomain interactions triggered by the cyclic nucleotide observed in wild-type R-subunit have been largely severed by the mutation W260A.

When bound to the C-subunit, the CNB domains of both mutant and wildtype unfolded at higher forces than in the apo state, 18.1 ± 3.0 pN for the mutant CNB-A domain and 14.4 ± 2.5 pN for the mutant CNB-B domain (Tables S1). The higher unfolding forces are likely the result of the stabilization of the CNB domain due to surface contacts with the C-subunit, which must be broken when the domains are mechanically unfolded. In contrast to the results observed in apo or cAMP-bound states, there were no discernable differences between the wild-type and the W260A mutant when bound to the C-subunit. This suggests that the mutation does not globally affect the formation of the inactive PKA holoenzyme. The negligible effect is possibly due to the position of W260 in the crystal structure of the PKA complex, where W260 contributes local electrostatic interactions with C-subunit (4).

### cAMP binding reshapes the folding energy landscape of the CNB-B domain

**Figure 2:**
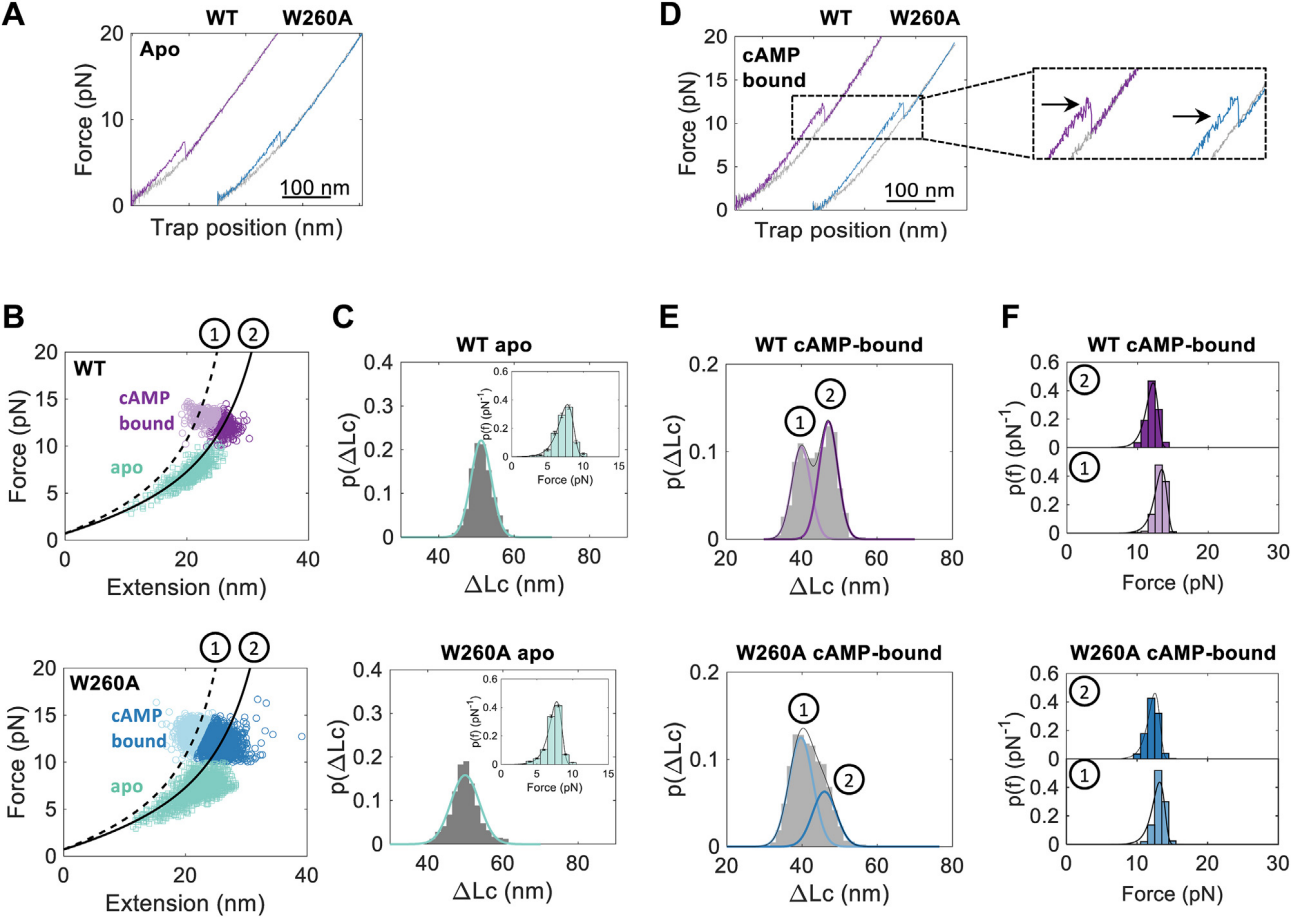
**Identification of two conformations in the unfolding events of the truncated CNB-B domain in a cAMP-bound state.***A*, force-extension curve of the unfolding of WT (*purple*) and W260A (*blue*) truncated CNB-B domain in apo state. Refolding event is shown in *gray*. *B*, WLC analysis for the truncated CNB domains in WT (*top*) and W260A (*bottom*) (Equation 3). Clustering analysis with Gaussian mixture model was performed to determine cluster assignment for the unfolding of the full-length CNB-B domain (aa. 243–376, *darker shade*, *solid line* labeled with “2”, ΔLc = 49 nm, *FD* = 3.3 nm) or the unfolding of the β-sandwich only of CNB-B domain (aa. 268–376, *lighter shade*, *dashed line* labeled with “1”, ΔLc = 40 nm, *FD* = 2.9 nm). Apo state is shown in *green square*. d_folded_ was measured from the crystal structure (PDB 1RGS). cAMP-bound state is shown in *purple* for WT and *blue* for W260A. *C*, probability distribution of the change in contour length for CNB-B domain in apo state. Inset corresponds to the force probability distribution. *D*, force-extension curves as in (*A*) but in the cAMP-bound state. Zoomed in displayed the unfolding events with more detail, where the N3A_B_ motif is pointed with an *arrow*. *E*, probability distribution of the change in contour length for CNB-B in cAMP-bound state. *Black line* indicates the fitted model from the clustering analysis. The *colored lines* correspond to each mixture component. *F*, probability distribution of the unfolding forces of CNB-B in cAMP-bound state. Fitted curves correspond to Equation 2. Color code and the numbering in (*C*) and (*E* and *F*) are the same as (*B*). CNB, Cyclic-nucleotide binding; WLC, Worm-like chain; WT, wild type.

Of the three functional states of the R-subunit investigated in this study, the cAMP-bound state showed the most significant mutational effects over τ_0_ and Δx^‡^, particularly the CNB-B domain. Changes in τ_0_ and Δx^‡^ can be attributed either to local destabilizing effects or long-range allosteric effects due to the loss of inter-domain interactions with CNB-A. We first investigated local mutational effects by characterizing the truncated mutant CNB-B domain with and without cAMP and compared its unfolding behavior and conformation to the WT domain (Fig. 2).

In the apo state, the WT and mutant CNB-B domains unfolded in a single cooperative event and at a similar F_ave_ (Fig. 2*A* and Table S2). Moreover, the observed change in contour length upon unfolding (ΔLc) of 50.1 ± 4.2 nm matched the expected value for the natively folded CNB-B domain according to the worm-like chain (WLC) model (Fig. 2*B*, green populations, and Experimental procedures, Equation 3.) In the presence of cAMP, the CNB-B domains unfold following two molecular trajectories: In one case the domain unfolds in a single step with a ΔLc = 49.9 ± 3.0 nm. In the second case, the domain unfolds in two consecutive steps with a ΔLc = 9.9 ± 2.7 nm and a ΔLc = 40.4 ± 2.1 nm (Fig. 2*D*, inset and Fig. 2*E*). This observation indicates that the presence of cAMP reshapes the folding energy landscape of the CNB-B domain, energetically decoupling the N3A_B_ motif and the β-subdomain to unfold in two steps instead of a single cooperative one. Interestingly, while the F_ave_ of the CNB domains bound to cAMP was higher than in the apo state, there were no differences between WT and the W260A mutant (Fig. 2*F* and Table S2). However, the fraction of trajectories in which the mutant CNB-B domain displays two unfolding steps (66%) is significantly higher than that of WT (48%), a mutational effect that is invariant to the refolding time between each pulling experiment (Table S3). Therefore, the mutation exacerbates the decoupling between the two structural elements in the CNB-B domain.

Previous structural studies on the CNB-A domain showed that the relative orientation of the N3A_A_ motif and the β-subdomain depend on cAMP binding, wherein the presence of cAMP moves the N3A_A_ motif away from the β-subdomain (3, 4). Our previous single molecule optical tweezers studies provided a thermodynamic foundation for such differences in conformation, in which we showed that the β-subdomain of the cAMP-bound CNB-A domain unfolds independent of its N3A_A_ motif (5). Here, the observed ΔLc = 9.9 ± 2.7 nm and 40.4 ± 2.1 nm for the cAMP-bound CNB-B domain match the size of the N3A_B_ motif and the β-subdomain, respectively (Fig. 1*A*, zoomed in). Therefore, the simplest interpretation of our results is that the response of the CNB-B domain to cAMP binding is similar to that of the CNB-A domain, where the N3A_B_ motif and the β-subdomain are energetically uncoupled and therefore unfold independent of each other.

### W260A destabilizes inter- and intra-domain interactions in the R-subunit

**Figure 3:**
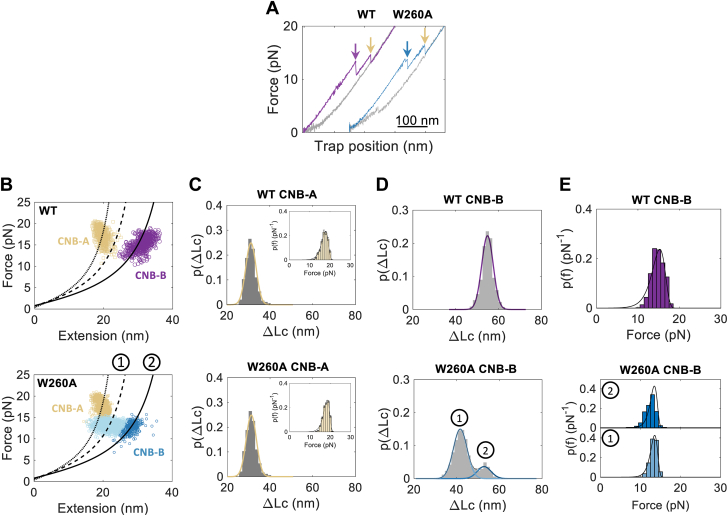
**Identification of two conformations in the unfolding events of CNB-B domain in the R-subunit.***A*, force-extension curve of the unfolding of WT (*purple*) and W260A (*blue*) R-subunit. Refolding event is shown in *gray*. The *arrows* point to the major unfolding event, where *purple* and *blue* points to the CNB-B domain and *yellow* points to the CNB-A domain. *B*, WLC analysis for the CNB domains in wildtype (*top*) and W260A (*bottom*) R-subunit bound to cAMP. Clustering analysis with gaussian mixture model was performed to determine cluster assignment for the unfolding of the full-length CNB-B domain (aa. 233–376, *purple* and *blue circles*, *solid line* labeled with “2”, ΔLc = 53 nm, *FD* = 4.3 nm) or the unfolding of the β-sandwich only of CNB-B domain (aa. 268–376, *light blue circles*, *dashed line* labeled with “1”, ΔLc = 40 nm, *FD* = 2.9 nm). CNB-A domain is shown in *gold square* (aa. 120–233, *dotted line*, ΔLc = 31 nm, *FD* = 1.2 nm)). d_folded_ was measured from crystal structures 1RGS. *C*, the Probability distribution of the change in contour length for the CNB-A domain. Inset corresponds to the force probability distribution. *D*, probability distribution of the change in contour length for CNB-B. *Black line* indicates the fitted model from the clustering analysis. The *colored lines* correspond to each mixture component. *E*, probability distribution of the unfolding forces of CNB-B. Fitted curves correspond to Equation 1. Color codes in (*C*–*E*) are the same as (*B*). CNB, Cyclic-nucleotide binding; WT, wild type.

Next, we investigated how the presence of the CNB-A domain allosterically affects the folding energy landscape of the CNB-B domain bound to cAMP (Fig. 3). WLC analysis of the unfolding rips of the WT and W260A mutant R-subunits revealed indistinguishable ΔLc and F_ave_ for the CNB-A domain (Fig. 3, *A*–*C* and Table S1). In contrast, the molecular trajectories of the mutant CNB-B domain mostly displayed two unfolding steps (80%, labeled as “1” and “2” in Fig. 3, *B* and *D*, bottom), whereas the WT domain exclusively unfolded in a single step (Fig. 3, *B* and *D*, top, and Table S3). Thus, the presence of the CNB-A domain in the WT protein provides the necessary interdomain contacts to energetically recouple the N3A_B_ motif to the CNB-B β-subdomain when bound to cAMP. Together with our findings on the truncated W260A CNB-B domain, the decoupling between the N3A_B_ motif and the β-subdomain cannot be overcome by the presence of the CNB-A domain in the mutant R-subunit. This interpretation agrees with the indistinguishable F_ave_ for the mutant CNB-B domain as truncation or as part of the R-subunit, which indicates lack of inter-domain contacts (Fig. 3*E*). Altogether, we show that the W260A mutation has a pronounced effect on the R-subunit residue networks by destabilizing both the interdomain interactions between the two CNB domains and intradomain interactions between the N3A_B_ motif and the β-subdomain.

### Allosteric coupling between W260A and N3A motifs

**Figure 4:**
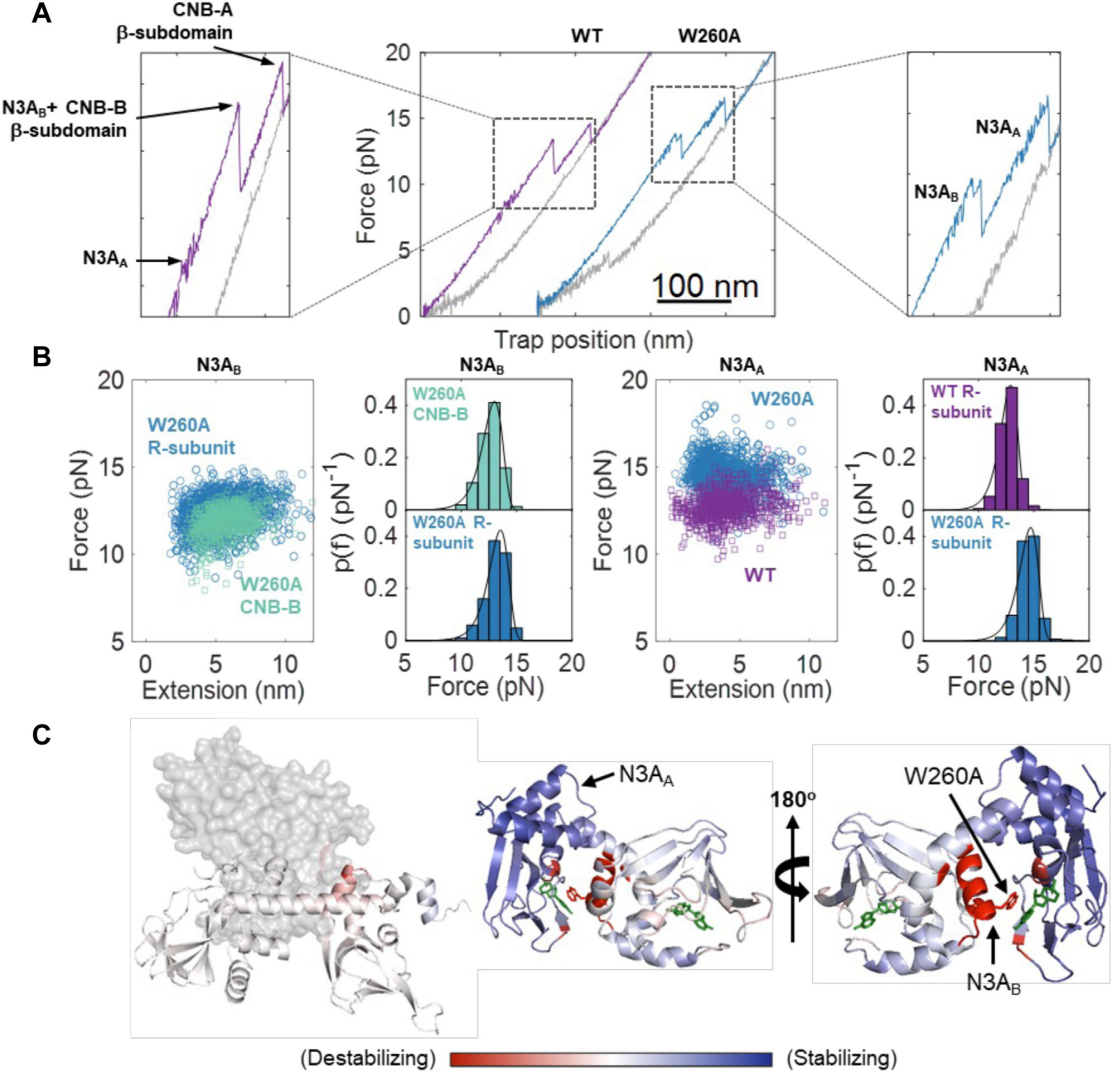
**Opposite effects on the N3A motifs stability due to W260A.***A*, *mid:* Force-extension curve of the unfolding of WT (*purple*) and W260A mutant (*blue*) in cAMP-bound states. This panel is the same as shown Figure 1*D*. *Left and right:* Zoomed-in force-extension curve to highlight the N3A motifs. *B*, *left to right:* WLC analysis of N3A_B_ motif in W260A R-subunit (*blue circle*) and in truncated W260A CNB-B domain (*green square*). Probability distribution of the unfolding forces of the respective comparison. WLC analysis of N3A_A_ in WT R-subunit (*purple square*) and W260A R-subunit (*blue circle*). Probability distribution of the unfolding forces of the respective comparison. *Solid line* corresponds to fitting Equation 1. *C*, allosteric effects due to W260A mutation in C-Subunit-bound state (*left*) and cAMP-bound state (*right*) by AlloSigMA. Color intensity corresponds to destabilization (−1.5 kcal/mol) to stabilization (+2.0 kcal/mol) due to mutation. *Black arrows* denote the W260A and the two N3A motifs. CNB, Cyclic-nucleotide binding; WLC, Worm-like chain; WT, wild type.

Given the widespread mutational effects of the W260A on the cAMP-bound R-subunit, we further investigated if the mutation exerts allosteric effects over the CNB-A domain itself. In previous mechanical unfolding studies, we have observed that the WT R-subunit unfolds in three transitions: the N3A_A_ motif unfolds first, followed by the CNB-B domain (N3A_B_ motif and its β-subdomain) and ending with the β-subdomain of CNB-A (Fig. 4*A*, left) (5). In the case of the mutant R-subunit, we observed four unfolding transitions, two for each CNB domain. The first two transitions had ΔLc of 11.3 ± 3.0 nm and 39.5 ± 3.3 nm that match the N3A_B_ motif and the CNB-B β-subdomain, respectively. The last two unfolding transitions had a ΔLc = 8.7 ± 2.5 nm that corresponds to the N3A_A_ motif, and a ΔLc = 30.4 ± 4.7 nm that matches the CNB-A β-subdomain. (Fig. 4*A*, right).

As expected for two non-interacting domains, like the CNB domains in the W260A R-subunit, the F_ave_ for the N3A_B_ motif was the same as those observed for its truncation, 12.79 ± 0.74 pN and 11.29 ± 0.83 pN, respectively (Fig. 4*B*, left). However, the N3A_A_ motif in the mutant R-subunit unfolded ∼2 pN higher than the WT at 14.5 ± 0.9 pN (Fig. 4*B*, right). Given that the two CNB domains in the W260A R-subunit do not establish inter-domain contacts (Fig. 1*D*, cAMP-bound), the most parsimonious interpretation for the observed stabilization of the N3A_A_ motif is long-range allosteric effects. To gain additional insight on long-range mutational effects, we used the software AlloSigMA (8, 9) to evaluate the mutational effect of W260A over the stability of residues in the R-subunit when bound to the C-subunit or to cAMP. In the C-subunit-bound state, the mutation exerted minimal overall perturbations on the R-subunit, a result that is consistent with our experimental observations showing no differences in unfolding forces between wildtype and mutant CNB domains (Fig. 4*C*, left). However, the calculations revealed major mutational perturbations throughout the R-subunit in the cAMP-bound state. First, the α-helix that harbors W260A mutation was substantially destabilized (Fig. 4*C*, in red), in agreement with the experimental results showing the N3A_B_ motif unfolding independently of its β-subdomain. Second, the entire CNB-A domain was stabilized by the mutation with the N3A_A_ motif displaying the strongest effect (Fig. 4*C*, in blue), which is consistent with our results where the N3A_A_ unfolds at higher forces than in WT. Altogether, the experimental and computational results revealed that, despite structural conservation, the N3A motifs experienced opposite effects due to the W260A mutation.

### W260A abolishes cooperative interactions during cAMP binding

**Figure 5:**
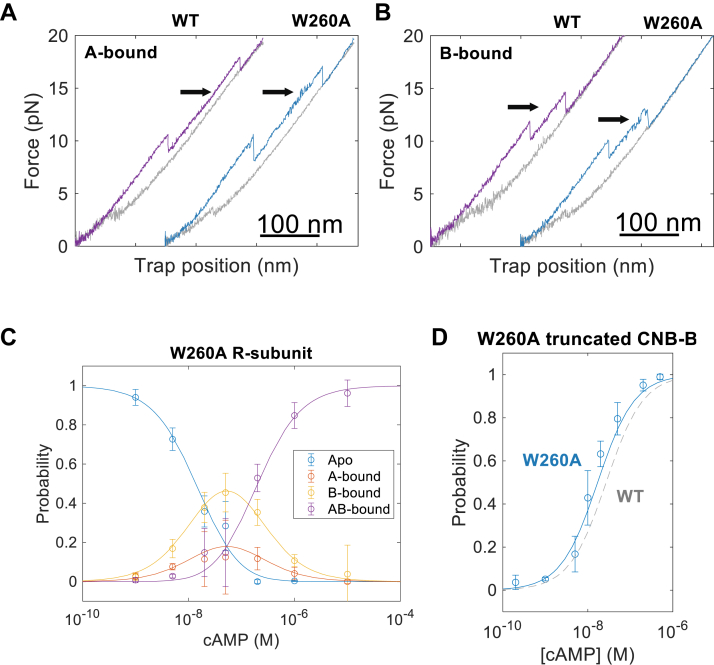
**Functional consequence of W260A during cAMP binding.***A*, force-extension curve of the unfolding of A-bound of the wildtype (WT, *purple*) W260A (*blue*). *Black arrows* point to the N3A motif when cAMP binds to either the CNB-A or CNB-B domain, which are only evident for W260A. *B*, force-extension curve of the unfolding of B-bound. *Colors* and *arrows* are the same as (*A*). Wildtype trajectories in (*A*) and (*B*) are adapted from Hao *et al.* (5)*. C*, cAMP titration plot showing the probability of apo, intermediate (A-bound or B-bound), and fully bound (AB-bound) states for W260A. The concentration range of cAMP is from 1 nM to 100 μM. *D*, cAMP titration plot of W260A truncated CNB-B domain from 0.2 nM to 0.5 μM. Error bars in panels (*C*) and (*D*) are the standard deviation from data of at least five different molecules for total of n = 3519 and 7612 unfolding events, respectively. *Dashed line* in (*D*) depicts the data fitting for the cAMP titration plot of WT previously published by Hao *et al.* (5). Data fitting (*solid lines*) in (*C*) and (*D*) are generated from *PyFolding*. Individual data points for the titrations in (*C*) and (*D*) are in the Source Data File accompanying this publication. CNB, Cyclic-nucleotide binding.

Because of the large-scale effects that the W260A mutation exerts over local or distant regions of the R-subunit, we investigated the functional consequences of the mutation in terms of cAMP binding affinities and cooperativities. We performed mechanical unfolding experiments with the mutant R-subunit under different cAMP concentrations to populate four distinct states: apo, intermediate states wherein only the CNB-A or the CNB-B domain is bound to cAMP (termed A-bound and B-bound, respectively), and the fully-bound state (termed AB-bound) states (Fig. 5). Due to the unique unfolding forces and ΔLc for each CNB domain when bound to cAMP, we were able to establish criteria that allowed us to assign each unfolding trajectory to one of the four possible states (Fig. 5, *B* and *C* and Supporting information).

In the A-bound state of wildtype, the CNB-B domain (N3A_B_ motif and β-subdomain) unfolds first followed by the CNB-A β-subdomain (Fig. 5*A*). The partial interdomain interactions triggered by cAMP binding to the CNB-A domain are insufficient to drive folding of the N3A_A_ motif (5). The W260A mutant in the A-bound state had a similar unfolding order but with a major difference: the N3A_A_ motif in the CNB-A domain displayed a clear unfolding transition after the CNB-B domain unfolds. This indicates that the N3A_A_ motif is stabilized by the W260A mutation, even in a singly cAMP-bound state. Due to the lack of interdomain interactions in W260A, we surmise that the N3A_A_ motif is in a different conformation compared to the WT protein, establishing stabilizing intra-domain contacts with the CNB-A β-subdomain.

In the B-bound state of WT, the N3A motifs and β-subdomains of both CNB domains unfold as single cooperative units, wherein the unbound CNB-A domain unfolds first (Fig. 5*B*). However, the W260A N3A_B_ motif remained decoupled from its β-subdomain, in agreement with what we observed from the unfolding of the AB-bound state (Fig. 4*A*, right) and the truncated cAMP-bound CNB-B domain (Fig. 2*D*).

Having characterized the unfolding trajectories of each cAMP-bound species at different cAMP concentrations, we built a single-molecule titration curve and globally fitted the population of each cAMP-bound species to determine cAMP binding affinity constants and cooperativity (Fig. 5*C*, Table S5 and Experimental procedures) (10). For the first cAMP binding event, both CNB domains have weaker binding affinities in the mutant than the WT: 3-fold difference for the CNB-A domain and 2-fold difference for the CNB-B domain. Moreover, while the WT R-subunit exhibits positive cooperativity on the second cAMP binding event (5), the mutant displayed a non-cooperative behavior (Table S5). The nearly identical affinity for the CNB-B domain in the R subunit and the truncated CNB-B domain confirms the lack of cAMP binding cooperativity.

The lack of cAMP binding cooperativity in the W260A R-subunit is likely due to the absence of inter-domain interactions in partially cAMP-bound states as seen in WT. For instance, the cAMP-free CNB domain in A- and B-bound states of WT exhibit a higher F_ave_ than the apo state but lower than the AB-bound state (5). In contrast, the F_ave_ between the cAMP-bound CNB domains in the mutant R-subunit and their respective cAMP-bound truncated domains are nearly identical (Tables S2 and S4).

### Structurally conserved CNB domains have unique thermodynamic signatures

**Figure 6:**
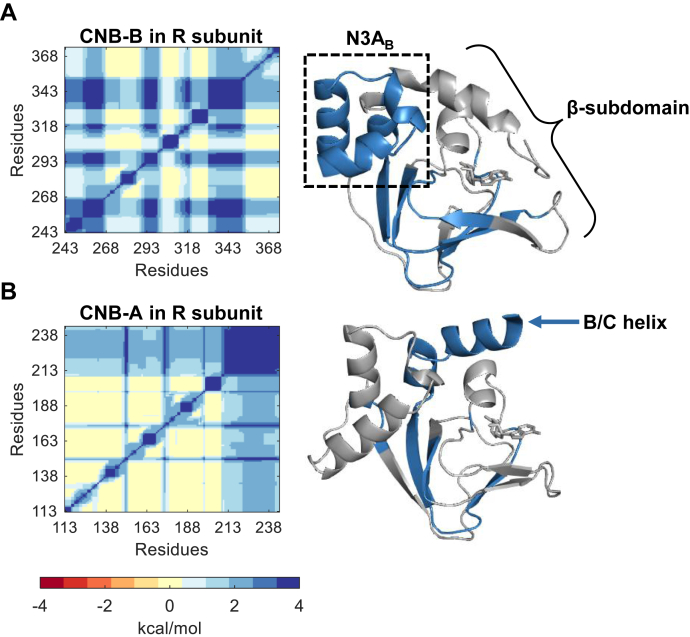
**Thermodynamic coupling of cAMP-bound R subunit.***Left*: Heatmap of the pairwise analysis of thermodynamic coupling of the CNB-B domain (*A*) and the CNB-A domain (*B*). The color scaling indicates negative coupling (−4 kcal/mol) in *red* to positive coupling (4 kcal/mol) in *blue*. A side-by-side comparison of the thermodynamic coupling of the CNB domains in the R subunit or as truncations bound to either the C-subunit or cAMP can be found in Fig. S2. *Right*: The positively coupled residues were mapped to the respective each CNB domain crystal structures (PDB: 1RGS). Positively coupled residues are found throughout the entire CNB-B domain, whereas in the CNB-A domain positive coupling is mostly observed in the B/C helix (*blue arrow*). CNB, Cyclic-nucleotide binding.

Despite the high structural similarity shared between the two CNB domains of PKA (residues 113–242 for the CNB-A domain and 243–379 for the CNB-B domain, RMSD = 0.8 Å for the cAMP-bound state and 0.7 Å for the C-subunit-bound state), the results obtained with optical tweezers show different conformational responses toward cAMP binding. Furthermore, we show that the W260A mutation produces both local and long-range allosteric effects across the CNB domains, and these effects are unique for each domain. These observations motivated us to ask whether each CNB domain in the PKA regulatory subunit has a unique network of thermodynamically coupled residues. Thus, even if the CNB domain structures look alike, their thermodynamic properties and response to mutations may be different. We used COREX/BEST, which is an algorithm that allowed us to calculate thermodynamic coupling as correlated thermodynamic fluctuations between each residue pair in computer-generated protein ensembles (Experimental procedures) (11). The results are described in a NxN matrix, where N is the total number of residues for each CNB domain (Fig. 6).

The thermodynamic coupling between the CNB-A and CNB-B domains in the same functional state (*i.e.*, cAMP-bound) display different patterns that map to different parts of their structure (Fig. 6). High thermodynamic coupling (colored in blue) was observed throughout the CNB-B domain, including the N3A_B_ motif and several β-strands in the β-subdomain (Fig. 6*A*). However, for the CNB-A domain, high thermodynamic coupling was largely localized to residues that conform the B/C helix (Fig. 6*B*, blue arrow). Importantly, differences in thermodynamic coupling between the CNB domains were also observed in the C-bound state (Fig. S2). These results support our hypothesis that despite having the same structure, the CNB domains have different thermodynamic signatures.

**Figure 7:**
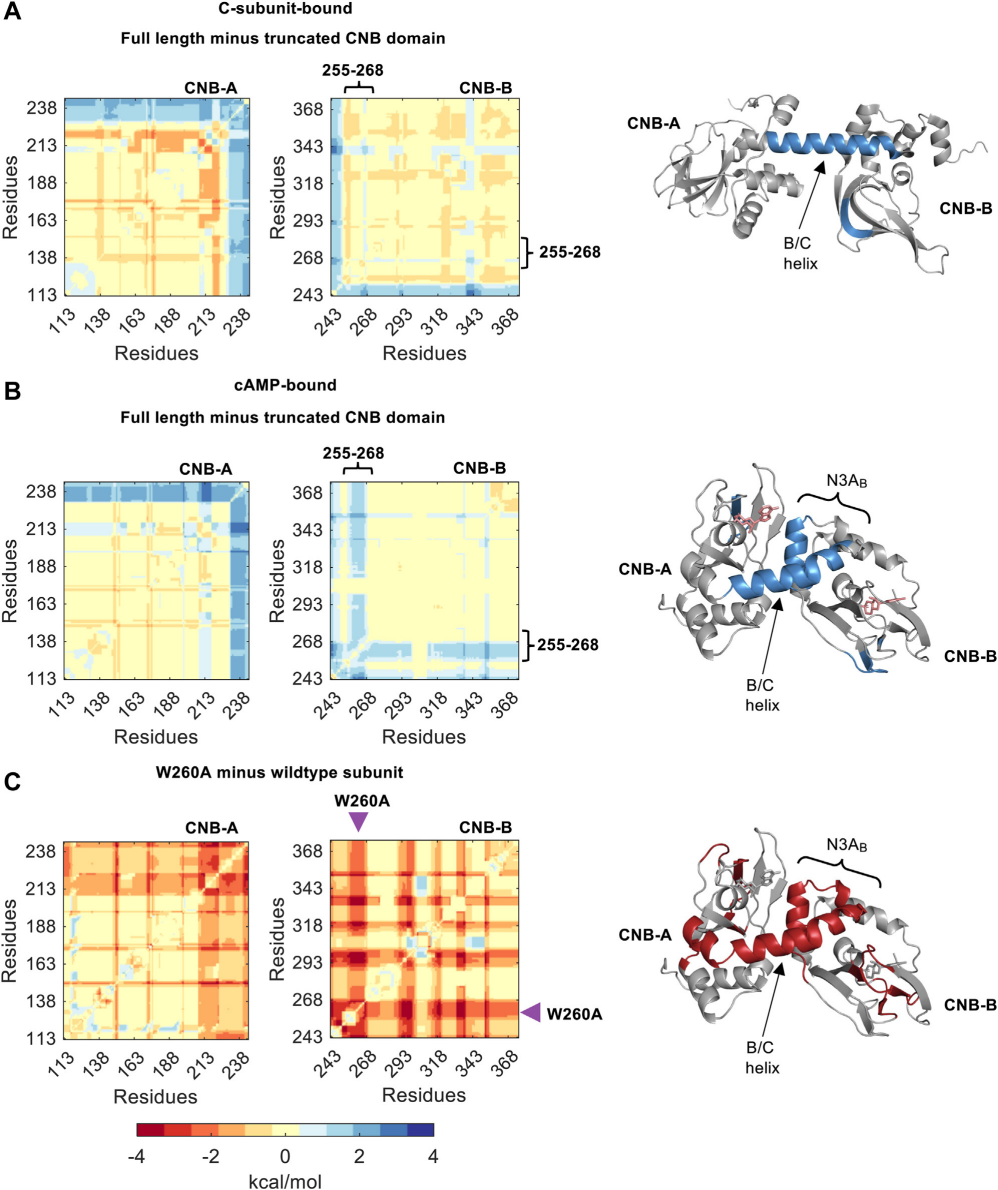
**Thermodynamic coupling of the PKA R-subunit in different functional states and the effect of W260A on the cAMP-bound state.***A*, effect of the coupling in the absence of CNB-B (*left*) or CNB-A (*right*) in C-bound state (PDB: 2QCS). *B*, effect of the coupling in the absence of CNB-B (*left*) or CNB-A (*right*) in cAMP-bound state (PDB: 1RGS). The plots in (*A*) and (*B*) correspond to ${\Delta \Delta G}_{j,k}^{full\;length}-{\Delta \Delta G}_{j,k}^{truncated\;domain}$. C-terminus of N3A_B_ motif (residues 255–268) display higher coupling to cAMP binding in the presence of the CNB-A domain, whereas the N3A_A_ motif (residues 120–150) or the N-terminus of the N3A_B_ motif (residues 233–254) display similar response in both functional states. *C*, comparison of WT and W260A in the cAMP-bound state. The plots correspond to ${\Delta \Delta G}_{j,k}^{W260A}-{\Delta \Delta G}_{j,k}^{WT}$. *Purple arrows* indicate the position of W260A mutation. The crystal structures highlight the positive coupling regions (>0.5, *blue*) in (*A*) and (*B*), respectively, or the negative coupling regions (<0.5, *red*) in (*C*). CNB, Cyclic-nucleotide binding.

Next, we dissected intradomain from interdomain thermodynamic coupling by performing the calculations on each CNB domain as truncations (*i.e.*, intradomain coupling) and as part of the R-subunit (*i.e.*, coupling due to interdomain interactions). The patterns for thermodynamic coupling of each CNB domain either as part of the R-subunit or as a truncated domain was overall similar, but in certain regions, there were noticeable differences (Fig. S2). Such differences are not unexpected as there is ample experimental evidence showing that CNB domains display allosteric behavior in multimeric complexes (*i.e.*, cAMP binding cooperativity) not only in PKA but in other proteins harboring CNB domains in their structure (12, 13, 14). To quantify these differences, we subtracted the thermodynamic coupling of each truncated CNB domain from the R-subunit. The difference maps were rendered in blue, red, and yellow representing higher, lower or no difference in thermodynamic coupling between the CNB domains in the R-subunit relative to the truncated domains (Fig. 7).

In the C-subunit-bound state, the presence of the neighboring CNB domain increases the thermodynamic coupling of residues 230 to 242 in the CNB-A domain, and residues 243 to 249 in the CNB-B domain. These two regions correspond to the B/C-helix that connects the two CNB domains in the R-subunit (Fig. 7*A*, cartoon). The high thermodynamic coupling exclusively localized to the B/C-helix indicates that the core structures of the CNB domains (*i.e.*, N3A motifs and β-subdomains) are largely uncoupled and independent from each other, in agreement with our experimental observations and results using AlloSigMA that show absence of long-range effects due to W260A in the C-bound state (Fig. 1, *D* and *E* bottom, and Fig. 4*C*, left, Experimental procedures, Equation 7).

In the cAMP-bound state, we also observed high thermodynamic coupling around the B/C-helix when the two CNB domains are together (Fig. 7*B*). However, an additional region between residues 255 to 268, belonging to the N3A_B_ motif, displayed high thermodynamic coupling in the R-subunit. The high coupling shared between the N3A_B_ motif, and the B/C-helix in the R-subunit indicates the formation of a new cAMP-dependent residue network that not only stabilizes inter-domain contacts with the CNB-A domain but also intra-domain contacts within the CNB-B domain itself. This interpretation agrees with our experimental results in which the N3A_B_ motif unfolds independent of the β-subdomain in the truncated CNB-B domain (Fig. 2*E*, top), whereas in the R-subunit the same domain always unfolds as a single cooperative unit and at a higher force (Fig. 3*D*, top).

### Global disruption of thermodynamically coupled residue networks by W260A

Given that the N3A_B_ motif in the cAMP-bound state displayed high thermodynamic coupling, it is likely that a mutation in this region, such as W260A, will have global effects over the R-subunit. Testing this hypothesis requires an experimentally determined high-resolution structure of the W260A R-subunit, which is not available. However, others have generated this mutant structure using molecular dynamics (MD) simulations (15). Before using the *in silico* mutant structure for our calculations, we compared the thermodynamic coupling of the WT cAMP-bound R-subunit before and after the same simulations that were used to determine the W260A structure. The difference would represent a background thermodynamic coupling due to the MD simulation. We find, however, that the two heat maps are very similar, and only after subtracting one heat map from the other, small differences in thermodynamic coupling are observed in limited regions of the protein (Fig. S3).

After accounting for the background thermodynamic coupling due to the MD simulation (Experimental procedures), the heat map of W260A revealed large differences compared to WT, both locally and long-ranged (Fig. 7*C*). The most significant effects were observed as loss of thermodynamic coupling in the N3A_B_ motif and the B/C helix, in agreement with our experiments showing that the CNB domains in the mutant R-subunit behave as independent domains (*i.e.*, no inter-domain interactions). In addition, loss of coupling due to W260A was observed in the β-subdomains of both CNB domains.

Interestingly, the large-scale mutant effects in the thermodynamic coupling that we observed emerged from differences in the relative orientation of the CNB domains and not because of differences in secondary structures between WT and W260A. For instance, aligning the CNB-A domain (residues 113–242) between the WT and the mutant resulted in a RMSD of 1.01 Å, likewise the alignment for the CNB-B domain (residues 243–376) resulted in 0.97 Å. However, a global alignment with both CNB domains had a RMSD = 5.7 Å. The disparity in RMSD is due to an 18.4-degree difference in rotation of the B/C helix at L233 in the mutant structure, which results in a 6.8 Å overall displacement for the CNB-B domain (Fig. S4). Altogether, the ensemble-based calculation was able to capture the mutational effects observed in experiments despite the similarity in structures.

## Discussion

### Differential mutational effects on each CNB domain in the PKA R-subunit

Conserved signaling domains have evolved to respond to external stimuli (*i.e.*, ligand binding or post-translational modifications), transduce a regulatory signal to other functional domains, and bind multiple protein partners (16). Such multifaceted functionality indicates that mutations may affect various features of a signaling domain, such protein assembly, ligand binding, or both. During the activation cycle of PKA, the CNB domains of the R-subunit experience large changes in conformation that are coupled to cAMP binding or assembly and disassembly with the C-subunit (2, 17). Previous studies have used different techniques to examine mutational effects in different functional states of PKA. For instance, fluorescent-based assays are commonly used to study PKA holoenzyme formation or dissociation or enzyme-coupled assays to measure C-subunit phosphorylation activity and infer cAMP binding affinity and cooperativity (4, 12, 18, 19). In contrast, in this study we used a single experimental technique, optical tweezers, to probe the R-subunit in various functional states and examine the mutational effects of W260A. By studying the effect of the mutation across these functional states, we obtained a holistic view on how a mutation causes differential defects on the enzyme’s function and regulation.

We find that W260A exerted differential effects on the folding energetics of the CNB domains depending on the functional state. Previous bulk studies reported that W260A disrupts interdomain interactions between the CNB domains (7, 20). Not only did we show the same local mutational effect on the R-subunit when bound to cAMP, but we also report long-range mutational effects on the CNB-A domain. Interestingly, the mutational effects were largely observed for the cAMP-bound state; effects in the C-subunit-bound state were almost inconsequential. The one-sided mutational effect is likely due to the role of W260 as a cAMP capping residue. When bound to the C-subunit, W260 is located near the N-terminal tip of the C-subunit activation loop (4). The minimal electrostatic interaction between the C-subunit and W260 likely results in indiscernible impact to the CNB domain structure and conformation (Fig. 4*C*, left). When bound to cAMP, W260 moves about 30 Å to stabilize the adenine ring of cAMP *via* π-π stacking. Such interaction is important for communication between the two CNB domains, which explains the significant mutational effect on the R-subunit when bound to cAMP.

### Structurally conserved CNB domains exhibit divergent cAMP response

Previous crystallographic and biophysical studies have focused on the importance of the N3A_A_ motif as a cAMP-dependent molecular switch (3, 5, 7). cAMP binding to the CNB-A domain facilitates the decoupling between the N3A_A_ motif and the β-subdomain. Here we revealed a previously undescribed structural response in the CNB-B domain when bound to cAMP that is similar, yet quantitatively different, than that of the CNB-A domain. Unlike the complete decoupling between the N3A_A_ motif and the CNB-A β-subdomain, in which these two subdomains always unfold independent of each other in the cAMP-bound state, the N3A_B_ motif of the cAMP-bound CNB-B domain fluctuates between two conformations, wherein the two subdomains are decoupled or interacting with each other. As a result, the truncated CNB-B domain bound to cAMP unfolds following two distinct pathways, either as a single cooperative unit or in two sequential steps. This dynamic conformational switching is reduced to a single state by the presence of the CNB-A domain in the WT R-subunit, where the CNB-B domain unfolding exclusively as a single cooperative unit.

Our results, therefore, show that CNB domains have evolved to not only respond to cAMP binding but also coordinate its conformational dynamics with the neighboring domain. Given that most CNB domain-containing proteins are multimeric, it is likely that our findings are conserved among other proteins like the HCN channel, cAMP receptor protein (CRP), or protein kinase G (PKG) where the CNB domains display coordinated structural changes upon cAMP binding (13, 14, 21, 22, 23).

### Effect of W260A on N3A motifs

A strength of using optical tweezers to investigate a mutational effect is that we can obtain information on local and long-range effects between non-contiguous protein domains or regions. This technique allows us to dissect the allosteric effects of a single mutation across the PKA R-subunit. Based on the structure, the mutation W260A directly affects interactions that are critical for cAMP binding to the CNB-A domain, which facilitates the conformational reorientation of the CNB domains. Therefore, we expected perturbations would arise from the location of the mutation, namely, loss of interdomain interactions. Less expected was observing the decoupling between the N3A_B_ motif and the CNB-B β-subdomain, where the mutation renders the N3A_B_ motif unable to lock in the final active conformation as in the WT protein. Moreover, the mutation also exerted long-range effects over the N3A_A_ motif, which unfolds at a higher force compared to wildtype (Fig. 4*A*). These long-range effects were captured by the computational analysis using AlloSigMA, which showed that the stabilized region within the R-subunit due to cAMP binding is highly concentrated to the N3A_A_ motif. All these results indicate that a single mutation can have an expansive effect across the protein, even on regions that are non-interacting.

### Functional defects due to W260A: beyond structures

Thus far, our experimental observations of the CNB domains in the W260A R-subunit indicate that they behave as two non-interacting domains tethered together, which is a behavior different from that observed in the WT protein. We therefore investigated if such a lack of inter-domain interactions would lead to no cooperativity during cAMP binding. We globally analyzed the cAMP titration data and determined a model with no cooperativity was sufficient to fit the data W260A R-subunit. Surprisingly, the binding affinities for cAMP of the mutant protein, both the truncated domains and the R subunit, were higher than the WT truncated CNB domains (Table S5). This result was particularly unexpected for the CNB-A domain given that the mutation is in the neighboring, non-interacting CNB-B domain. The apparent activation constant of W260A was 4.6-fold lower than the value of the WT with the Hill coefficient of 0.9 (4). Our findings suggest that the difference in the activation constant is due to the combination of binding affinity and conformational defects. These results indicate that the molecular origin and interplay between affinity and cooperativity is more complex than an initial structural view of two non-interacting domains linked together.

### Same structures, different thermodynamic properties

From the single molecule optical tweezers experiments, we established differential effects of the CNB domains in response to a binding partner, that is, cAMP or the C-subunit. The computational analysis of thermodynamically coupled residues further revealed that the CNB domains have unique interaction networks (Fig. 6). Previous studies have shown allosteric pluripotency in PKA due to divergent allosteric responses of the CNB domains (20, 24). These differential responses likely emerge from their unique network of thermodynamically coupled residues. Within these networks, we show that the coordinated response of the CNB domains to interactions with the C-subunit or cAMP binding largely stem from the B/C-helix (Fig. 7, *A* and *B*). In the C-subunit-bound conformation, several residues identified as highly coupled in the B/C-helix (R230, L233, M234, T237, and L238) interact with the C-subunit *via* hydrogen bonds and van der Waals interactions (3). In the cAMP-bound conformation, not only residues in the B/C helix but also amino acids belonging to the N3A_B_ motif displayed high coupling, agreeing with our experimental results.

Using the WT and W260A mutant R-subunit structures from MD simulations, we determined that the W260A mutation caused an 18.4-degree rotational difference starting at L233 of the B/C-helix. This rotational difference of the B/C-helix may be key to the destabilization between the CNB domains. The increase in flexibility correlates well with the increase in conformational dynamics observed by Guo and Zhou in molecular dynamic simulations (15) and the loss of interdomain coupling by Akimoto *et al.* (7) using NMR. Our studies further show that the re-orientation of the CNB-B domain leads to an expansive effect on thermodynamic coupling across the R-subunit (Fig. 7*C*). These results emphasize the role that the B/C-helix plays in interdomain communication beyond residues at the interface of the two CNB domains.

### Consequences on PKA activation and regulation cycle

**Figure 8:**
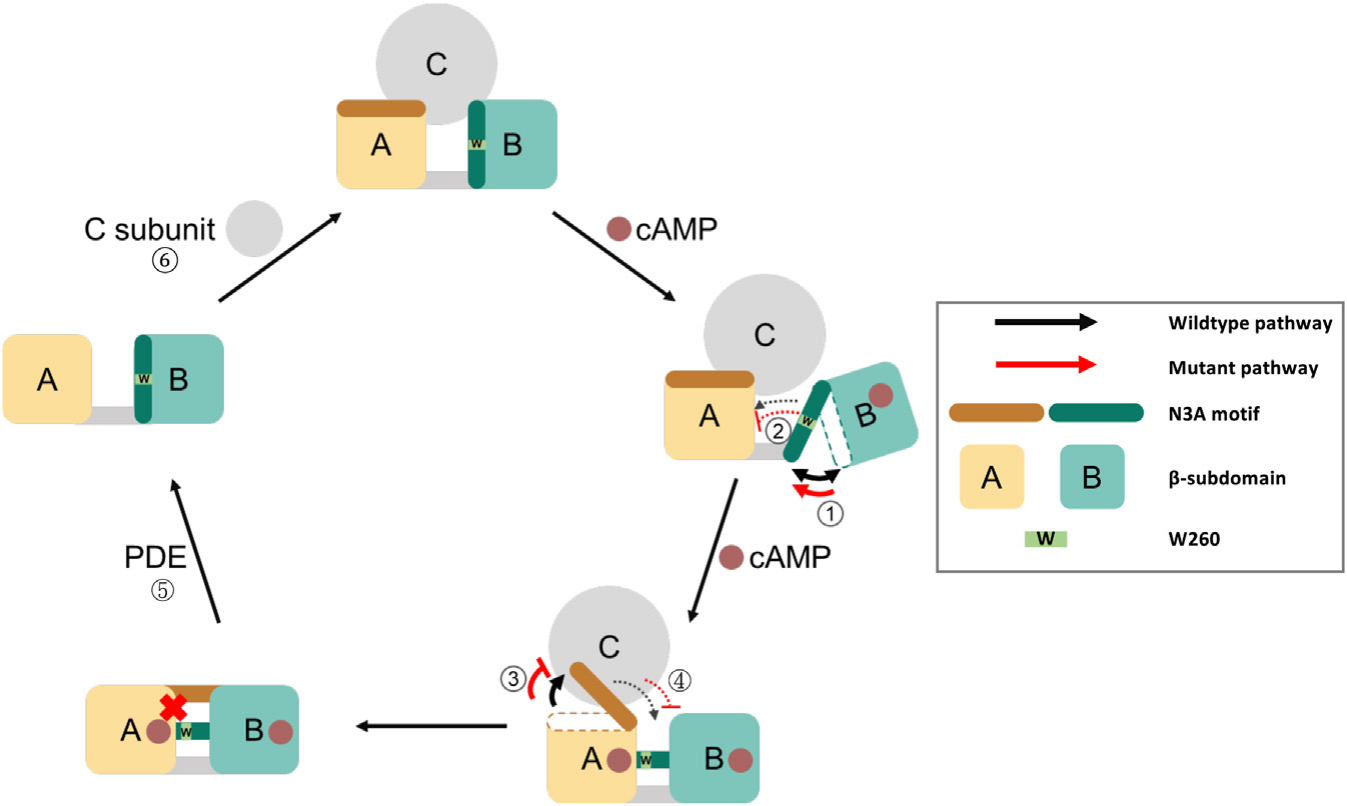
**The effect of W260A on PKA activation and regulation cycle.** A schematic of the activation pathway of the PKA C-subunit by cAMP. Starting with the inactive complex, each cAMP binding event triggers conformation changes to the R-subunit. The *black arrow* shows the pathway of the wildtype R-subunit, whereas the *grey arrow* shows the pathway of the W260A R-subunit. Conformational changes on the N3A motifs are indicated by the *curved arrows*, where *black curved arrows* correspond to WT R-subunit and *red curved arrows* correspond to the W260A R-subunit. Changes from the *bold arrows* trigger the subsequent movement in the *dashed arrows*. PKA, protein kinase A.

We provide a comprehensive computational and experimental study on the mutational effect of W260A to the PKA R-subunit in apo, C-subunit-bound, and cAMP-bound states. Figure 8 shows six steps (labeled ① to ⑥) in the activation cycle of PKA, in which we identified the consequences of W260A. In the WT R-subunit, cAMP binds first to the CNB-B domain, where the N3A_B_ motif becomes thermodynamically decoupled from the β-subdomain, thereby oscillating between two conformations (①). When the second cAMP binds to the CNB-A domain, the N3A_B_ motif stabilizes the cyclic nucleotide and becomes thermodynamically coupled with the CNB-B β-subdomain, behaving as a single cooperative unit (②). The binding of the second cAMP also leads to the destabilization of the N3A_A_ motif, breaking inte-domain interactions with the CNB-A β-subdomain domain and the C-subunit (③). As the C-subunit dissociates, the N3A_A_ motif stabilizes by establishing contacts with the CNB-B domain and the B/C helix (④). The activation cycle of PKA can be repeated *via* the removal of cAMP by phosphodiesterase (PDE, ⑤) and the rebinding of the C-subunit with ATP and Mg^2+^ (⑥). We show that W260A affects the activation pathway of PKA in three different steps: W260A shifts the fluctuation of the N3A_B_ motif towards a decoupled conformation (step ①), there are no inter-domain interactions due to the absence of W260 that serves as cAMP capping residue for the CNB-A domain (step ②), and, W260A stabilizes the N3A_A_ motif allosterically, possibly by retaining intra-domain interactions with the CNB-A β-subdomain (steps ③ and ④). These deficiencies in the conformation of the R-subunit due to W260A prevents the protein from achieving the final, cAMP-bound conformation observed for the WT protein.

The combination of experimental and computational analysis reveals that structurally conserved signaling domains have subtle thermodynamic differences and widespread effects due to a mutation. These unique behaviors may emerge from the fact that the CNB domains have evolved to behave as molecular switches, changing in conformation to adapt to the environment, be it a mutation or interacting with a binding partner. Going beyond the CNB domains in the PKA regulatory subunit, the single molecule optical tweezers approach in conjunction with the computational description of protein fluctuations presented in this study can be extended to understand the allosteric effects of mutations in other CNB domain-containing proteins and multi-domain assemblies (25, 26, 27).

## Experimental procedures

### Protein expression and purification

The pRSET plasmid containing the gene of regulatory subunit R1α residue 71 to 379 was used to create the W260A mutant *via* site-directed mutagenesis. The same plasmid was also used to create the truncated CNB-B domain (residue 243–379) and its mutated counterpart. Cysteine residues were modified on residues Y120C/S376C or M243C/S376C on the R-subunit and the truncated CNB-B domain, respectively, for the covalent attachment of double stranded (ds) DNA that serve as molecular handles. The protocols for the purification of PKA R1α, the truncated CNB domains, and the PKA catalytic subunit were previously published (5, 28, 29). See Supporting information for additional detail.

### Attachment of dsDNA handles to protein constructs

The protocol for dsDNA handle attachment as described previously (27). Briefly, the purified R-subunit or truncated CNB-B domain was covalently modified with dsOligos of 24-base pairs (bp) *via* thiol chemistry. A functional selection step using a homemade cAMP-coupled resin was used to remove unreacted DNA handles and to separate binding-competent DNA-protein chimeras from other by products, namely, misfolded or unfolded proteins. Before optical tweezers experiments, an aliquot of the eluted chimera was ligated with 370-bp dsDNA functionalized with biotin and digoxigenin. See Supporting information for more detail.

### Optical tweezers measurements and analysis

Protocols for force-ramp experiments with cAMP or with C-subunit were performed as previously published (7, 29). The microfluidic chamber was equilibrated with two different buffers depending on the experiment. Buffer 1 (50 mM Tris, 100 mM NaCl, 10 mM DTT, pH 7.6) was used for experiments in the apo or cAMP-bound state. Buffer 2 (10 mM MOPS, 50 mM NaCl, 1 mM MgCl_2_, 0.2 mM ATP, pH 7.0) was used for experiments with PKA C-subunit at 100 nM. The main difference between the two buffers was the presence of MgCl_2_, ATP, and C-subunit, which were critical for the formation of the inactive complex (C-subunit-bound state). The incubation time for protein samples with 3.1 μm anti-digoxigenin coated beads is 15 min with and without cAMP, and 30 min with the C-subunit. The sample mixture is diluted to 1 ml with the same buffer before applying to the microfluidic chamber. The concentration of the DNA–protein chimera in the microfluid chamber is calculated to be below the picomolar range.

Force-ramp experiments were conducted at a constant pulling velocity of 75 nm/s, with 10 s refolding time at 1.5 pN unless specified in the text. For example, the refolding time was varied between 1 and 10 s for WT and mutant R-subunits or truncated CNB-B domains to assess the dynamics of the N3A_B_ motif unfolding (Table S3). For each experimental condition, at least 400 traces were collected from 3 to 5 different molecules.

The unfolding events were analyzed using a custom-built MATLAB program to obtain unfolding forces and the associated extension changes. The distribution of unfolding forces was fitted using Bell’s model to obtain the rate of unfolding at zero force (*k*_*0*_) and the distance to the transition state in the unfolding reaction (*Δx*^*ǂ*^) (30):

(1) $$p\left(F\right)=\frac{k\left(F\right)}{r}e^{\frac{k_{0}}{{\Delta x}^{ǂ}r}}$$

where

(2) $$k\left(F\right)=k_{0}e^{\frac{F{\Delta x}^{ǂ}}{k_{B}T}}$$

The lifetime *(τ)* at zero force is calculated from the inverse of *k*_*0*_. See Supporting information for more details on the analysis of optical tweezers data analysis.

The WLC model was used to determine the number of residues involved in each unfolding event:

(3) $$F=\frac{k_{B}T}{p}\left[\frac{1}{4}{\left(1-\frac{\left(\Delta x+FD\right)}{L_{c}}\right)}^{-2}-\frac{1}{4}+\frac{\left(\Delta x+FD\right)}{L_{c}}\right]$$

where *p* is the persistence length of the polypeptide (0.65 nm) (31), Δx is the change in molecular extension upon unfolding, folded distance (FD) is the distance between the residues with DNA handles in the folded state, and Lc is the contour length, which is calculated by multiplying the number of amino acids (aa) by 0.365 nm/aa (32). We report changes in the contour length (ΔL_C_) by subtracting the FD between the attachment points in the folded protein from L_C_.

### AlloSigMA

The structured-based statistical mechanical model of allostery (SBSMMA) was used in this study. SBSMMA evaluates the allosteric communication upon perturbations such as ligand binding and/or mutations. Here, allosteric effects of the PKA R-subunit bound to the C-subunit or to cAMP due to the W260A mutation were quantified using the AlloSigMA webserver (http://allosigma.bii.a-star.edu.sg/). The methodology was described by Guarnera *et al.* (8, 9).

The crystal structures of the R subunit bound to the C-subunit or cAMP (PDBs 2QCS, 1RGS), as well as truncation CNB-B domain (extracted from each PDB) were used to define a harmonic model of the unperturbed (*i.e.*, WT) and perturbed (*i.e.*, mutant) states of the protein domain. The energy function for the unperturbed state is given by

(4) $$E_{r\to r^{0}}^{WT}=\sum_{i,j}k_{i,j}{\left(d_{i,j}-d_{i,j}^{0}\right)}^{2}$$

where r and r_0_ are the coordinates of the protein structure in generic and reference configurations, respectively; k_i,j_ is the spring constants of the protein harmonic model, and d_i,j_ and d_i,j_^0^ are inter-residue distances. The mutated protein is defined as

(5) $$E_{r\to r^{0}}^{MUT}=\sum_{i\neq MUT,j}k_{i,j}{\left(d_{i,j}-d_{i,j}^{0}\right)}^{2}+\alpha \sum_{j}k_{MUT,j}{\left(d_{i,j}-d_{i,j}^{0}\right)}^{2}$$

where the second term accounts for the effect of a point mutation. α is a perturbation parameter, which acts as a scaling factor of the force constant and reflects the type of mutation considered in the perturbation. If α = 1, the energy function of the mutated protein is identical to that of the WT. A weak α indicates that the mutation interacts weakly with the rest of the protein, whereas a strong α indicates strong interactions between the mutation and the rest of the protein. The allosteric potential of the protein evaluates the elastic work that is exerted on a residue as a direct result of the protein’s dynamics:

(6) $$U_{i}\left(\sigma \right)=\frac{1}{2}\sum_{\mu }\varepsilon_{\mu ,i}\sigma_{\mu }^{2}$$

$\varepsilon_{\mu ,i}$ depends on the normal modes of the wildtype and mutated proteins ($\varepsilon_{\mu ,i}^{WT}$ and $\varepsilon_{\mu ,i}^{MUT}$, respectively), and the coefficients σ_μ_ are Gaussian variables with the variance 1/ε_μ,i_. The per-residue free energy changes are calculated from the configurational ensembles for the WT and mutated residues. The ensemble of a single residue is characterized by all possible displacements assumed by the neighboring residues:

(7) $$\Delta G_{i}^{MUT}=\frac{1}{2}k_{B}T\sum_{\mu }\frac{\varepsilon_{\mu ,i}^{MUT}}{\varepsilon_{\mu ,i}^{WT}}$$

AlloSigMA considers two extreme scenarios: the over-stabilizing mutation (termed up mutation) and the contact-eliminating mutation (termed down mutation). The difference between the up and down mutations gives rise to the overall allosteric effect.

### COREX

We used the COREX algorithm to investigate the long-range thermodynamic coupling between residue pairs in the R subunit and the truncated CNB domains (residues 113–242 for the CNB-A domain and residues 243–376 for the CNB-B domain). COREX generates a native-state ensemble from the target protein structure through the combined unfolding of adjacent groups of residues defined as folding units, which are treated as native or as unfolded peptides. The free energy of each conformational state within the ensemble is calculated with a surface-area parametrization that has been validated experimentally (33, 34). The calculations were performed on C-subunit-bound and cAMP-bound R-subunit structures (PDB 2QCS and 1RGS, respectively), and molecular dynamic simulation structures of WT and W260A R-subunit bound to cAMP were generously provided by Prof. Huan-Xiang Zhou, University of Illinois Chicago (see Reference (15) for details on molecular dynamic simulation methods). Briefly, the simulated structures used in this study were obtained from three simulation averages, each one corresponding to the last 50 ns of a 150 ns simulation, sampling every 4 ps.

For each structure (experimental or simulated), we extracted the associated change in free energy of paired residues (ΔΔG_*j,k*_). ΔΔG_*j,k*_ represents the long-range effect of the paired residues over each other. The result is visualized in a color-coded N x N matrix, where N is the residue number of the protein. See Supporting information for details on the calculation of thermodynamic coupling. The same analysis was performed with the truncated CNB domains. The truncated CNB domain structures were extracted from 1RGS and 2QCS and stored as a separate ∗.pdb file. By subtracting the truncated CNB domains from the R-subunit (${\Delta \Delta G}_{j,k}^{R\;subunit}-{\Delta \Delta G}_{j,k}^{truncated\;domain}$), we can identify regions with high coupling in the presence or absence of the neighboring CNB domain. Data analysis and visualizations were performed in MATLAB.

## Data availability

All data needed to evaluate the conclusions in the paper are present in the paper and/or the Supporting information and in Source Data file.

## Supporting information

This article contains supporting information (2, 3, 5, 10, 11, 29, 34, 35, 36, 37, 38, 39, 40).

## Conflict of interest

The authors declare that they have no conflicts of interest with the contents of this article.
