# Supplementary material for: Conformational changes in protein kinase A along its activation cycle are rooted in the folding energetics of cyclic-nucleotide binding domains
Source: J Biol Chem. 2023 May 6;299(6):104790. doi: 10.1016/j.jbc.2023.104790 (PMC10279917; doi:10.1016/j.jbc.2023.104790)
Supplement: Supporting information [file mmc1.docx]

Conformational changes in PKA along its activation cycle are rooted in the folding energetics of cyclic-nucleotide binding domains

Amy K. Chau^1^, Katherine Bracken^1^, Lihui Bai^1^, Dominic Pham^1^, Lydia Good^1^, Rodrigo A. Maillard^1,*^

^1^ Department of Chemistry, Georgetown University, Washington, DC 20057, USA

^*^ Corresponding author: Email: Rodrigo.Maillard@georgetown.edu

**This PDF file includes:**

Supplementary Text

Figs. S1 to S4

Tables S1 to S5

Supplementary Text

**Expression and purification of PKA regulatory subunit**

The Regulatory (R) subunit or the truncated CNB domains was expressed in BL21 (DE3) pLysS at 37 ^o^C until an OD of 0.6. Isopropyl β-D-1-thiogalactopyranoside (IPTG) was added in a final concentration of 0.5 mM at 18 ^o^C overnight. Cells were harvested by centrifugation at 5000 rpm for 20 min at 4 ^o^C and resuspended with a ratio of 1:10 w/v lysis buffer (20 mM MES, 100 mM NaCl, 2 mM EGTA, 2 mM EDTA, 5 mM DTT, pH 6.5) supplemented with inhibitors (10 mM Benzamidine, 0.4 mM 4-(2-Aminoethyl)-benzenesulfonyl fluoride (AEBSF), 1 mM Pepstatin, 1 mM Leupeptin, 28 mM Tosyl-phenylalanyl-chloromethyl-ketone (TPCK), 28 mM Tosyl-L-lysylchloromethane-ketone (TLCK) and 10 mM 3-isobutyl-1-methylxanthine (IBMX). Cells were lysed with a microfluidizer at 10000 psi.

The homogenized mixture was separated by centrifugation at 15000 rpm for 40 min at 4 ^o^C. The supernatant, where the protein of interest is found, was treated with 40 % ammonium sulfate for 1 hour at 4 ^o^C to precipitate the soluble proteins. The precipitate was collected at 10000 rpm for 10 min at 4 ^o^C, then solubilized with lysis buffer followed by another centrifugation at 16000 rpm for 10 min at 4 ^o^C to remove remaining contaminants. The supernatant was added to an agarose-based resin functionalized with cAMP and incubated overnight at 4 ^o^C. Protein was eluted from the resin with excess cGMP and separated by size-exclusion chromatography (HiLoad 16/600 Superdex 200 pg, Cytiva)) via FPLC (Bio-Rad) to remove unbound nucleotides and protein aggregates. The purified protein was stored in storage buffer (50 mM MES, 200 mM NaCl, 2 mM EGTA, 2 mM EDTA 5 mM DTT, pH 5.8) with 25 % (v/v) glycerol at -80 ^o^C.

**Expression and purification of PKA catalytic subunit**

The catalytic (C) subunit with an N-terminal his-tag was expressed in *E. coli* Rosetta 2 (DE3) competent cells (EMD Millipore) with 0.5 mM IPTG overnight at 18 °C. The cells were lysed in C-subunit lysis buffer (20 mM Imidazole, 30 mM MES, 50 mM KCl, 1 mM EDTA, and 5 mM DTT, pH 6.5) and lysed with a microfluidizer at 10,000 psi. The cell lysate was centrifuged at 15000 rpm for 1 hour at 4 °C. The spun supernatant was batch bound to 20 mL of nickel agarose resin and eluted with 1 M imidazole. The purified C-subunit was stored in 30 mM MES, pH 6.5, 50 mM KCl, 1 mM EDTA, 5 mM DTT, 1 M imidazole and 30 % (v/v) glycerol. Prior to optical tweezers experiment, the purified protein was buffer exchanged and filtered using a 0.2 µm cellulose acetate membrane syringe filter equilibrated with PKA activity buffer (10 mM MOPS, pH 7.0, 50 mM NaCl, 1 mM MgCl_2_, 0.2 mM ATP).

**dsDNA handle attachment**

The R-subunit or the truncated CNB domains were modified with two cysteine residues in each construct to serve as the DNA handle attachment positions. The two sets of dsOligo consist of a thiol-group at the forward 5’-end and a phosphate-group at the reverse complimentary 5’-end. The thiol-group reacts with cysteines on the protein whereas the phosphate group reacts with dsOligo that are functionalized with either biotin or digoxigenin to form complete tweezers-ready tethers (Fig. 1C). Purified protein is dialyzed against crosslinking buffer (50 mM Tris, 100 mM NaCl, 10 mM DTT, pH 7.6) at 4 ^o^C overnight to remove glycerol and to reduce the cysteine residues. At the same time, thiol-modified dsOligo are reduced with a final concentration of 10 mM DTT overnight at 37 ^o^C. The next day, protein was concentrated to at least 5 mg/mL. The excess DTT was removed by three Micro Bio-Spin columns (Bio-Gel P6, Bio-rad) preequilibrated with crosslinking buffer. Protein was reacted with excess 2,2’-dithioldipyridine (DTDP) for 2 hours at room temperature. The excess DTDP was removed by three Micro Bio-Spin 6 columns. Excess DTT from both dsOligo was removed by three Micro Bio-Spin 6 columns and immediately reacted with DTDP-activated protein at 1:1:1 ratio with approximately 100 µM each. The reaction was incubated at 4 ^o^C overnight.

The crude reaction was incubated with cAMP-coupled resin (~25 µL) for a minimum of 4 hours at 4 ^o^C to remove unreacted dsOligo and to select for functional proteins. The dsOligo-protein chimera-bound resin was washed with 20 column volumes of crosslinking buffer. The chimera was eluted with 0.02 mM, 0.2 mM, 2 mM, and 20 mM cAMP in crosslinking buffer at increasing concentration. Each elution step included 30 minutes of incubation. All elution fractions were aliquoted separately with 25-30 % glycerol and stored at -80 ^o^C.

**Tweezers data analysis**

Data analysis was performed using a custom-built MATLAB program (*32*). Changes in extension as a function of force were analyzed with the worm-like chain (WLC) model in order to determine the number of amino acid residues involved in the unfolding event. The WLC model is used to describe the force-dependent behavior of a flexible polymer under an applied force (*36*):

$$F=\frac{k_{B}T}{p}\left[ \frac{1}{4}\left( 1-\frac{(\Delta x+FD)}{L_{c}} \right)^{-2}-\frac{1}{4}+\frac{(\Delta x+FD)}{L_{c}} \right]$$

where *p* is the persistence length of the polypeptide (0.65 nm) (*32*), ∆x is the change in molecular extension upon unfolding, *FD* is the distance between the residues with DNA handles in the folded state, and L_c_ is the contour length, which is calculated by multiplying the number of amino acids (aa) by 0.365 nm/aa (*33*). For protein constructs where there is more than one unfolding event, we used the WLC model, the crystal structure of the R-subunit (*2*, *3*), and Monte Carlo simulations to estimate the structural elements or domains involved in the unfolding event. See the section “CNB domain assignments for each functional state” for more detail.

The distribution of unfolding forces was fitted using the model developed by Dudko *et al*. (*31. 37-38*) to obtain kinetic unfolding parameters The following equation is applied to extract the force dependent unfolding rate from the folding force histograms:

$$p\left( F \right)=\frac{k\left( F \right)}{r}*e^{\frac{k_{0}}{{\Delta x}^{ǂ}r}}*e^{-\left[ \frac{k\left( F \right)}{{\Delta x}^{ǂ}r} \right]\left[ 1-\left( \frac{vF{\Delta x}^{ǂ}}{{\Delta G}^{ǂ}} \right)^{1-\frac{1}{v}} \right]}$$

where *r* is the force loading rate, *k_0_* is the rate of unfolding at zero force, *F* is the force, *Δx^ǂ^* is the distance to the transition state from the folded to the unfolded state. *ΔG^ǂ^* is the free energy of activation in the absence of external force. *ν* is the scaling factor that specifies the nature of the underlying free-energy landscape (*6*). *k(F)* is the force-dependent unfolding rate, which equals to

$$k\left( F \right)=k_{0} \left( 1-\left( \frac{vF{\Delta x}^{ǂ}}{{\Delta G}^{ǂ}} \right)^{\frac{1}{v}-1}*e^{{\Delta G}^{ǂ}\left[ 1-\left( 1-\frac{vF{\Delta x}^{ǂ}}{{\Delta G}^{ǂ}} \right)^{\frac{1}{v}} \right]} \right)$$

The lifetime *(τ)* at zero force is obtained by taking the inverse of *k_0_*. When *ν* = 1, the model is simplified to the Bell’s model. Errors of *τ_0_* and *Δx^ǂ^* were obtained by bootstrapping. The dataset was resampled to the number of data points in the original dataset. In the resampling process, each unfolding force that is drawn is subsequently replaced in the pool of data, so it is possible to sample each point more than once. *τ_0_* and *Δx^ǂ^* were determined for each resampling dataset. The process was repeated for 100 times to obtain the mean and standard deviation for each parameter.

**CNB domain assignments to each unfolding event or rip in the apo, cAMP-bound and C-subunit bound states**

In this study, we examined the unfolding of the R subunit in three functional states: apo, cAMP-bound, and C-subunit bound. For each functional state, we used different methods to assign the CNB domains to specific unfolding events seen in the optical tweezers’ trajectories.

Due to the similar unfolding forces and changes in extension of the two rips in the wildtype R-subunit in the apo state, we performed Monte Carlo simulation with the experimental data from the truncated CNB domains. The extracted folded state lifetime at zero force and distance to the transition state from the truncated CNB domains were used to simulate the unfolding trajectories in the R-subunit. Based on previous published studies ( 5), we determined that the CNB domains in the R subunit in the apo state do not interact. This is based on the experimental observation that the unfolding energy landscape parameters of the CNB domains is the same as truncated domains or in the R subunit (5). The detailed procedures for the simulations can be found in the section “Monte Carlo simulation”.

The simulated unfolding forces of the CNB domains were compared against the unfolding forces of the experimental results of the wildtype R-subunit (Fig. S1A). The simulated result revealed that 80 % of the unfolding of R-subunit began with the CNB-B domain, followed by the CNB-A domain. The remaining sample size of 20 % accounted for the reverse order of the CNB-A domain unfolding first. Despite having a mixture of unfolding event, the F*_ave_* of the simulation and the experimental results were in agreement of each other.

We applied a similar process of analysis on the W260A mutant R-subunit in apo state (Fig. S1B). Because the mutation was in the CNB-B domain, we performed the force-ramp experiment using the truncated CNB-B domain and extracted the unfolding kinetic parameters. Using the unfolding energy landscape parameters of the wildtype CNB-A and the W260A CNB-B domain, we performed Monte Carlo simulations that showed a quantitative agreement with the experiments using the W260A R subunit, and thus, enabled us to parse out unfolding events that corresponds to each CNB domain (Fig. S1B).

The existing cAMP-bound R subunit crystal structure (PDB: 1RGS) and previously analysis on the wildtype protein (5) provide a foundation for analyzing the unfolding trajectories of the W260A R subunit. Because the unfolding trajectories of the W260A R subunit displayed additional complexity compared to the wildtype R subunit, we implemented clustering for the CNB-B domain to distinguish the unfolding of the N3A_B_ motif and the CNB-B β-subdomain separately or together. See the section “Clustering ΔLc by Gaussian Mixture Model for the W260A CNB-B domain” for more details.

Lastly, for unfolding trajectories the C-subunit bound state, the unfolding forces of the CNB domains were similar. In addition to the crystal structure (PDB: 2QCS), we implemented a clustering method to the WLC analysis to systematically assign an unfolding event to a specific CNB domain. See the section “WLC clustering analysis for C-subunit bound R subunit” for more detail.

**Monte Carlo simulations**

The dynamic trajectory of a single tether was carried out by stochastic Monte Carlo simulations (*39*). A similar analysis was performed in previous single-molecule optical tweezers studies on PKA (*5*, *30*). Briefly, the simulations were performed by discretization of simulation time into small units ∆t, such that transition probabilities within a given time step were < 0.05. For the simulations in this study, ∆t was chosen to be 5 ms, or at 200 Hz. Within each time step, iteration of the following processes permitted physical simulation of polymer unfolding:

(1) Calculation of force-extension for WLCs in series: For each polymer unit in the tension chain (i.e., DNA and protein), a force-extension curve is calculated to relate the polymer unit’s fractional extension to applied forces between 0 and 20 pN. At each force, the total extension of the tension chain is the sum of the products of fractional extension and contour length for each polymer unit within the tension chain. Parameters for WLC calculations are provided in a separate paragraph below.

(2) Stretching of WLCs in series: At each time step, the tension force exerted by the polymer chain is balanced by the pulling force exerted by the optical trap plus a random fluctuating force, $\vec{F_{chain}}\left( t \right)+\vec{F_{trap}}\left( t \right)+\vec{\zeta}\left( t \right)=0$, where ∆(t) is a random number chosen from a zero-mean normal distribution with standard deviation $\sigma=\sqrt{\frac{2k_{B}T\gamma_{0}}{\Delta T}}$. The Stokes’ drag coefficient, $\gamma_{0}=6\pi r\eta$, was calculated as a spherical 2.1 μm diameter bead with radius, r = 1.05 μm, in a medium with dynamic viscosity, η = 1 cP. The solution to the above force equation was solved numerically at each time step, which also by extension directly calculated the total extension of tension chain and position of the bead in the trap.

(3) Protein unfolding transition probabilities: If protein unit A in the tension chain is folded, it is converted to an unfolded state with probability $P\left( A \right)=k_{A}exp\left[ F(t)\Delta\frac{x_{A}^{\ddagger}}{k_{B}T} \right]$. Similarly, if protein unit B in the tension chain is folded, it is converted to an unfolded state with probability $P\left( B \right)=k_{B}exp\left[ F(t)\Delta\frac{x_{B}^{\ddagger}}{k_{B}T} \right]$.

(4) Movement of the trapped bead: The trapped bead is held in an optical trap with Hookean spring constant, κ = 0.075 pN/nm. Throughout the simulation, the trap position is moved at a rate of 75 nm/s, therefore at each time step, the trap position is incremented by $x\left( t \right)=x\left( t-1 \right)+v\Delta T$.

(5) Time evolution: Simulation time, t, was incremented by Δt.

(6) WLC and other parameters used in simulations: 700 bp of DNA was simulated in the tether with a persistence length of P_DNA_ = 50 nm. The unfolded domains’ change in contour lengths are ∆Lc_CNB-A_ = 46 nm and ∆Lc_CNB-B_ = 52 nm with a persistence length of P_Protein_ = 0.65 nm. The folded state lifetime at zero force and the distance to the unfolding transition states were determined experimentally from the wildtype and W260A truncated CNB domains.

Discrete time Monte Carlo simulations were repeated for the same number of replicates as in the experimental results using MATLAB, and the features from the resulting stochastic trajectories were plotted directly.

**Clustering ΔLc by Gaussian Mixture Model for the W260A CNB-B domain**

A Gaussian Mixture Model was implemented in the clustering analysis of ΔLc (Fig. 2E and Fig. 3D). This model provided assignment for each data point to a clustering group, which we used to identify and quantify the major unfolding event for the truncated CNB-B domain and the CNB-B domain in the R-subunit. Briefly, a matrix consisting of force, change in extension, and converted ΔLc was fed into a script with the model. The model implements the expectation-maximization (EM) algorithm to generate clusters based on ΔLc. Bayesian Information Criteria (BIC) score (*40*) was used to evaluate the optimal number of clusters present in the dataset, which results in two clusters for the CNB-B domain when bound to cAMP. Each cluster set was extracted into its own matrix to evaluate the unfolding force distributions (Fig. 2F and Fig. 3E). The analysis was repeated five times for each dataset to ensure convergence.

**WLC clustering analysis of unfolding trajectories of the R subunit bound to the C subunit.**

To assign observed unfolding rips to a structural element of the R-subunit bound to the C-subunit, we first considered all possible pairs of contiguous rips through which the PKA R-subunit could unfold. Using the sequence and a crystal structure of the R-subunit (PDB: 2QCS), we calculated the contour length, Lc, and folded distance, *FD*, between the endpoint residues of each possible unfolding rip.

We iterated through all possible unfolding models, each defined by a pair of WLC curves representing the unfolding of N- and C-terminal regions of the PKA R-subunit, which differ in length in each model but are continuous and cover the entire length of the R-subunit in every model. We selected the best-fitting unfolding model for the data by minimizing the sum of the squared distances from all data points (i.e., unfolding rips) to the curves to which they were assigned. The data assignment to the curves similarly minimized the sum of the absolute distances between the WLC curves and all assigned data points, in this case while maintaining the condition that one observed rip from each unfolding trajectory must be assigned to each WLC curve in the model. Each trajectory reflects the unfolding of the entire PKA R-subunit, which is composed of both an N-terminal and a C-terminal unfolding unit, so one unfolding rip must represent the unfolding of each part of the protein. In practice, this meant that we iterated through each unfolding trajectory, tested both ways of assigning the two unfolding rips to the two WLC curves, and selected the assignment that minimized the sum of distances between the two points and their assigned WLC curves. This method gave us both the unfolding model for the R-subunit that best fit the data as well as an optimized assignment of the data points to the WLC curves representing the unfolding behavior specified by the model.

We also analyzed the combined ΔLc of both rips and confirmed that they correspond to the unfolding of the entire R-subunit. The same analysis was performed with the W260A mutant construct, and the combined ΔLc were the same as the that in the wildtype. This indicated the W260A mutant has no effect on the overall fold of the R-subunit.

**Fitting of cAMP titration data**

A single-molecule titration curve was generated for the analysis of cAMP binding to the W260A R-subunit. The *PyFolding* package was used with slight modification to globally fit the four cAMP-bound states with shared parameters. The script can be obtained at: <https://github.com/quantumjot/PyFolding> (*10*). Analysis using this module with the wildtype R-subunit was published previously (*11*). We established criteria to assign each of the unfolding trajectories of the W260A R subunit at various cAMP concentrations. The two end-states, apo and fully bound (termed AB-`bound), are directly characterized from experiments in which the cAMP concentration is zero or saturating at 1 mM, respectively (i.e., [cAMP] >> Kd of cAMP (5)). This direct characterization provides unique unfolding forces and ΔLc for apo and AB-bound states as follows:

1. Apo (A_0_B_0_): Both rips unfold below ~ 12 pN, with 45 nm < ΔLc < 49 nm.
2. AB-bound (A_1_B_1_): Rip 1 unfolds above 12 pN with ΔLc = 40 nm and rip 2 unfolds above 14 pN with ΔLc = 31 nm. The values of ΔLc are smaller than those observed in apo because the N3A motifs of each CNB domain unfold separately from the larger β-subdomains.

When we used concentrations of cAMP between 1nM and 100 uM, we observed two additional unfolding trajectories that have unfolding forces and ΔLc different from those obtained for apo and AB-bound states. These two additional trajectories were assigned to A-bound and B-bound based on the following:

1. A-bound (A_1_B_0_): Rip 1 unfolds below 10 pN with ΔLc = 49 nm and rip 2 unfolds above 14 pN with ΔLc = 31 nm. Rip 2 has an unfolding force and ΔLc indistinguishable from the truncated CNB-A domain (5). Rip 1 unfolds at a force and ΔLc characteristic of an apo CNB domain. Given that Rip 2 is assigned to the CNB-A bound to cAMP, Rip 1 must be, by process of elimination, the apo CNB-B domain.
2. B-bound (A_0_B_1_): Rip 1 unfolds at 11 pN with ΔLc near 45 nm and rip 2 unfolds above 13 pN with ΔLc between 40 or 49 nm. Here, rip 1 unfolds as the apo CNB-A domain, and rip 2 unfolds as the assigned CNB-B domain in AB-bound. It is important to note that the CNB-B domain ΔLc is 40 nm at 80 % occurrence and 49 nm at 20 % occurrence. We accounted for this variation during the assignments.

Having established the identity of each unfolding trajectory to a specific state (apo, A-bound or A_1_B_0_, B-bound or A_0_B_1_, and AB-bound or A_1_B_1_) a titration curve of each cAMP-bound state was plotted with the error bar showing the standard deviation of the dataset between > 5 different molecules for a total N = 3519. We use the following binding polynomial between cAMP and the two binding sites of the regulatory subunit:

|  |  | *k­_1_* |  |  |
| --- | --- | --- | --- | --- |
|  | A_0_B_0_ | $\rightleftharpoons$ | A_1_B_0_ |  |
| *k­_2_* | $\rightleftharpoons$ |  | $\rightleftharpoons$ | *k­_3_* |
|  | A_0_B_1_ | $\rightleftharpoons$ | A_1_B_1_ |  |
|  |  | *k­_4_* |  |  |

The relationship between cAMP concentration and each state are described below:

$$A_{0}B_{0} =\frac{1}{\left( 1 + k_{1}\left[ cAMP \right]+ k_{2}\left[ cAMP \right]+k_{1}k_{3}\left[ cAMP \right]^{2} \right)}$$

$$A_{1}B_{0}= \frac{k_{1}\left[ cAMP \right]}{\left( 1 + k_{1}\left[ cAMP \right]+ k_{2}\left[ cAMP \right]+ k_{1}k_{3}\left[ cAMP \right]^{2} \right)}$$

$$A_{0}B_{1}= \frac{k_{2}\left[ cAMP \right]}{\left( 1 + k_{1}\left[ cAMP \right]+ k_{2}\left[ cAMP \right]+ k_{1}k_{3}\left[ cAMP \right]^{2} \right)}$$

$$A_{1}B_{1}= \frac{{k_{1}k_{3}\left[ cAMP \right]}^{2}}{\left( 1 + k_{1}\left[ cAMP \right]+ k_{2}\left[ cAMP \right]+ k_{1}k_{3}\left[ cAMP \right]^{2} \right)}$$

**Ensemble-based modeling using COREX**

We used the COREX algorithm to investigate the thermodynamic coupling of the CNB domains in different functional states (*35, 41*). Briefly, COREX generates an ensemble of conformational states of the cAMP-bound and C-subunit bound R-subunits through an iteration of partitioning the protein sequence into groups of amino acid residues defined as folding units. Folding units are treated as native-like or as unfolded peptides. The probability of any given conformational state *i*, *P_i_*, is calculated from the free energy of the conformational state.

$$P_{i}=\frac{\exp\left( -\frac{\Delta G_{i}}{RT} \right)}{\sum_{i=0}^{N} \exp\left( -\frac{\Delta G_{i}}{RT} \right)}$$

The denominator is the sum of all possible states in the ensemble from the partitioning. The resulting free energy change reflects the relative stability of each residue is the ratio of the probability of a given residue is in the folded state (P*_f,j_*) over the probability of that same residue is in the unfolded state (P*_u,j_*):

$${\Delta G}_{f,j}=-RTln\left( \kappa_{f,j} \right)=-RT ln\left[ \frac{P_{f,j}}{P_{u,j}} \right]$$

The thermodynamic coupling of a residue pair is evaluated as the effect of an energetic perturbations of the residues over each other. For each residue, the perturbation energy considers the probabilities of states where residues *j* and *k* are both folded ($P_{f,j|f,k}$), both unfolded ($P_{nf,j|nf,k}$), or one in each state ($P_{f,j|nf,k}$ or $P_{nf,j|f,k}$):

$${\Delta G}_{f,j}^{pert,k}=-RTln\left( \kappa_{f,j}^{pert, k} \right)=-RTln\left( \frac{\varphi^{pert,k}P_{f,j|f,k}+P_{f,j|nf,k}}{\varphi^{pert,k}P_{nf,j|f,k}+P_{nf,j|nf,k}} \right)$$

The perturbation on residue *k* be stabilizing, destabilizing, or no effect over residue *j*. This thermodynamic effect can be quantified by:

$${\Delta\Delta G}_{f,j}^{pert,k}={\Delta G}_{f,j}-{\Delta G}_{f,j}^{pert,k}$$

It is important to note that this analysis does not require residues *j* and *k* to be in close proximity in the primary sequence or in the tertiary structure. Thus, ${\Delta\Delta G}_{f,j}^{pert,k}$ characterizes the long-range effects of a residue over another.

Given that the long-range perturbation may not be reciprocated equally, we consider a bidirectional thermodynamic coupling as the sum of the perturbation effect of residue *j* over *k* and the perturbation effect of residue *k* over *j*:

$${\Delta\Delta G}_{j,k}={\Delta\Delta G}_{f,j}^{pert,k}+{\Delta\Delta G}_{f,k}^{pert,j}$$

Thermodynamic coupling between two residues can be positive (ΔΔG*_j,k_* > 0), negative (ΔΔG*_j,k_* < 0), or neutral (ΔΔG*_j,k_* = 0). Positive coupling occurs when stabilization or destabilization of the residues are synchronous, negative coupling occurs when stabilization or destabilization of the residues are synchronous with opposite effects, and neutral indicates that the two residues are not thermodynamically coupled. We this analysis, we can investigate the long-range effects of the presence of a neighboring domain ( ${\Delta\Delta G}_{j,k}^{CNB,truncated}$ vs ${\Delta\Delta G}_{j,k}^{CNB,R subunit}$) or a W260A mutation (${\Delta\Delta G}_{j,k}^{WT}$ vs ${\Delta\Delta G}_{j,k}^{W260A}$).

**Reference for Supporting Information**

2. Y. Su, W.R.G. Dostmann, F.W. Herberg, K. Durick, N. Xuong, L.T. Eyck, et al., Regulatory subunit of protein kinase A: structure of deletion mutant with cAMP binding domains, *Science*, 269, 1995, 807–813.

3. C. Kim, N.-H. Xuong and S.S. Taylor, Crystal structure of a complex between the catalytic and regulatory (RIα) subunits of PKA, *Science*, 307, 2005, 690–696.

5. Y. Hao, J.P. England, L. Bellucci, E. Paci, H.C. Hodges, S.S. Taylor, et al., Activation of PKA via asymmetric allosteric coupling of structurally conserved cyclic nucleotide binding domains, *Nat. Commun.*, 10, 2019, 3984.

10. A.R. Lowe, A. Perez-Riba, L.S. Itzhaki and E.R.G. Main, PyFolding: open-source graphing, simulation, and analysis of the biophysical properties of proteins, *Biophys. J.*, 114, 2018, 516–521.

11. J. Vertrees, P. Barritt, S. Whitten and V.J. Hilser, COREX/BEST server: a web browser-based program that calculates regional stability variations within protein structures, *Bioinformatics*, 21, 2005, 3318–3319.

29.J.P. England, Y. Hao, L. Bai, V. Glick, H.C. Hodges, S.S. Taylor, et al., Switching of the folding-energy landscape governs the allosteric activation of protein kinase A, *Proc. Natl. Acad. Sci. U. S. A.*, 115, 2018, E7478–E7485.

34.V.J. Hilser and E. Freire, Structure-based calculation of the equilibrium folding pathway of proteins. correlation with hydrogen exchange protection factors, *J. Mol. Biol.*, 262, 1996, 756–772.

35.M.D. Wang, H. Yin, R. Landick, J. Gelles and S.M. Block, Stretching DNA with optical tweezers, *Biophys. J.*, 72, 1997, 1335–1346.

36.O. Dudko, G. Hummer and A. Szabo, Intrinsic rates and activation free energies from single-molecule pulling experiments, *Phys. Rev. Lett.*, 96, 2006, 108101.

37.C.A. Pierse and O.K. Dudko, Kinetics and energetics of biomolecular folding and binding, *Biophys. J.*, 105, 2013, L19–L22.

38.N.G.V. Kampen, *Stochastic Processes in Physics and Chemistry*, Third Edition, 2007, Elsevier; North Holland.

39.G. Schwarz, Estimating the dimension of a model, *Ann. Stat.*, 6, 1978, 461–464.

40.V.J. Hilser, D. Dowdy, T.G. Oas and E. Freire, The structural distribution of cooperative interactions in proteins: analysis of the native state ensemble, *Proc. Natl. Acad. Sci. U. S. A.*, 95, 1998, 9903–9908.


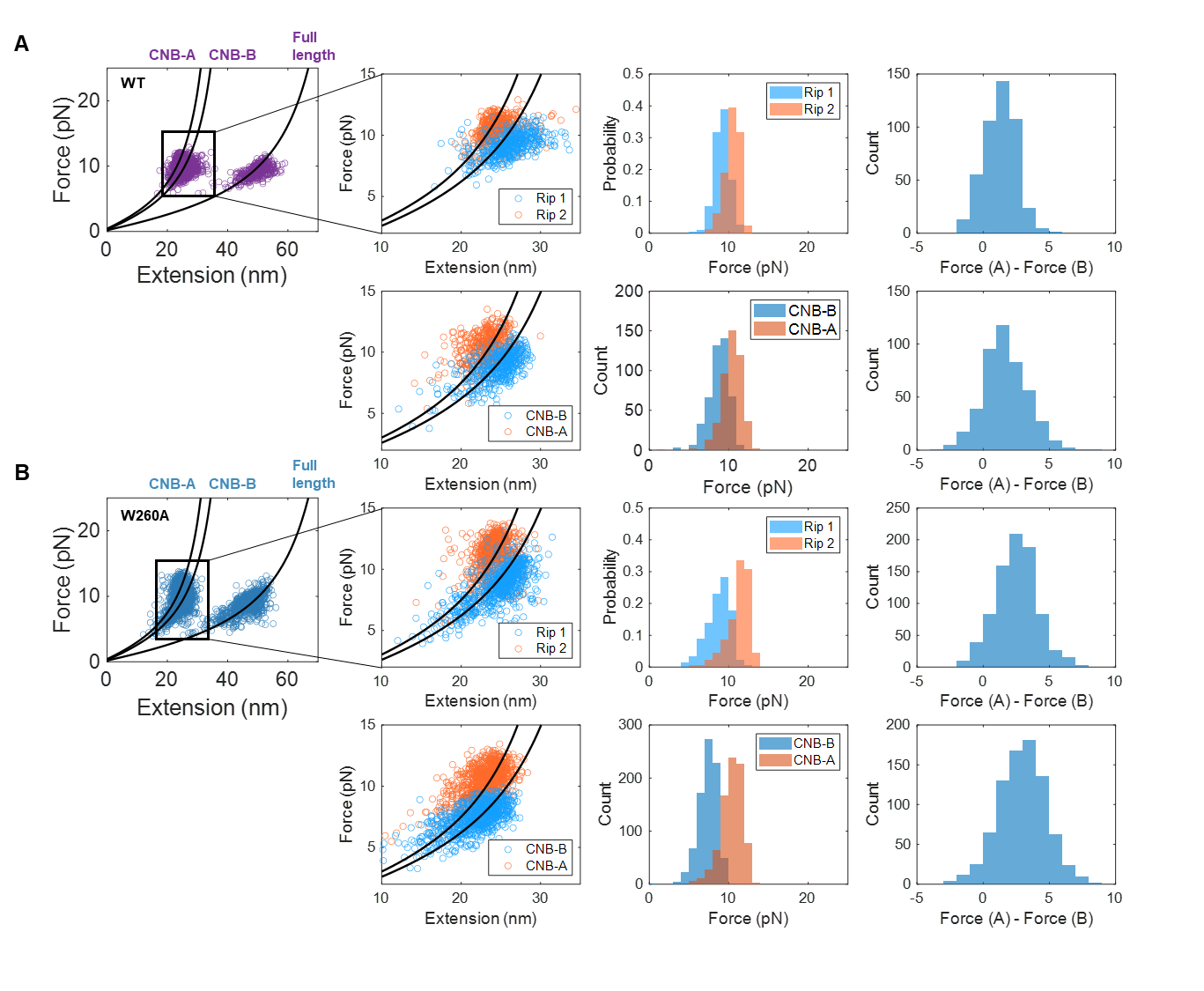


Fig. S1. Monte Carlo simulation of the unfolding of the CNB domains. WLC analysis for the CNB domains and full-length in the wildtype (A) and W260A (B) R-subunit in apo state. The black lines correspond to WLC models using Equation 1 in the main text: For the CNB-A domain (residues 120-242, ΔLc = 43 nm, *FD* = 2 nm), CNB-B domain (residues 243-376, ΔLc = 48 nm, *FD* = 1.5 nm), and for the full-length (CNB-A + CNB-B) (residues 120-376, ΔLc = 94 nm, *FD* = 4 nm). Experimental results from the respective CNB domains in apo state was used to simulate the unfolding of both CNB domains in a single polypeptide. The simulated results of rip 1 and rip 2 (top) were compared against the experimental results of the unfolding of the R subunit (bottom). Results were fitted with the WLC model and the corresponding unfolding forces were shown in a histogram. The distribution of Force A (orange) - Force B (blue) indicates the second unfolding event is at a higher force most of the time. Parameters of the truncated CNB domains in apo state were obtained from England *et al* (28)*.*


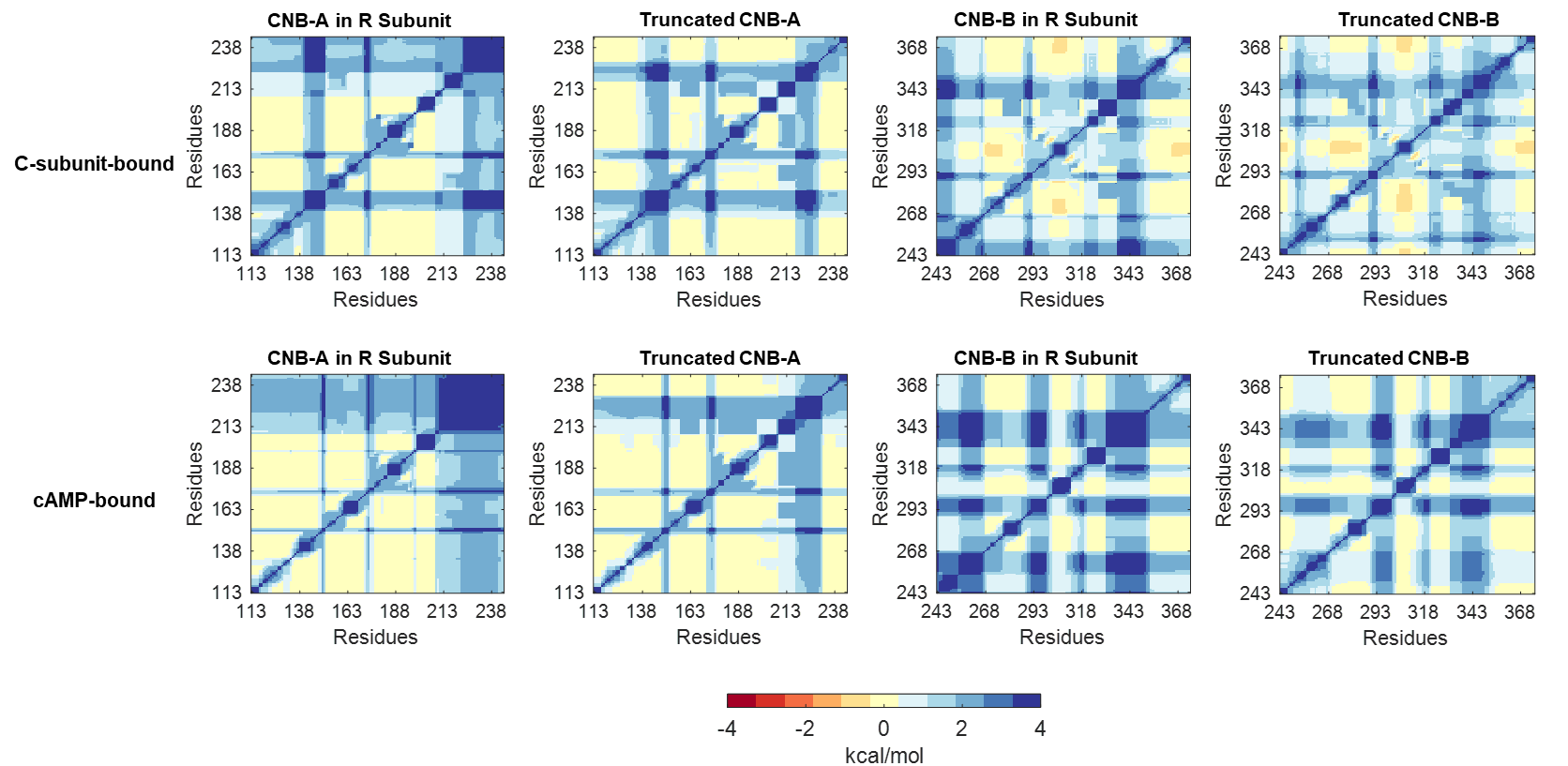


Fig. S2. Thermodynamic coupling of CNB domains in truncation or in the R-subunit. Using the COREX approach, thermodynamic coupling between each residue pair were plotted in a heat map. Each CNB domain were calculated as a truncated domain and as part of the R-subunit when bound to the C-subunit (top) or to cAMP (bottom). The color scaling represents the thermodynamic coupling between residue pair, from -4 kcal/mol (red) to 4 kcal/mol (blue). Residues displaying minimal thermodynamic coupling are colored in yellow. cAMP-bound CNB-A in R subunit and CNB-B in R subunit are reused in Fig. 6 to visualize the thermodynamic coupling with their respective crystal structures.


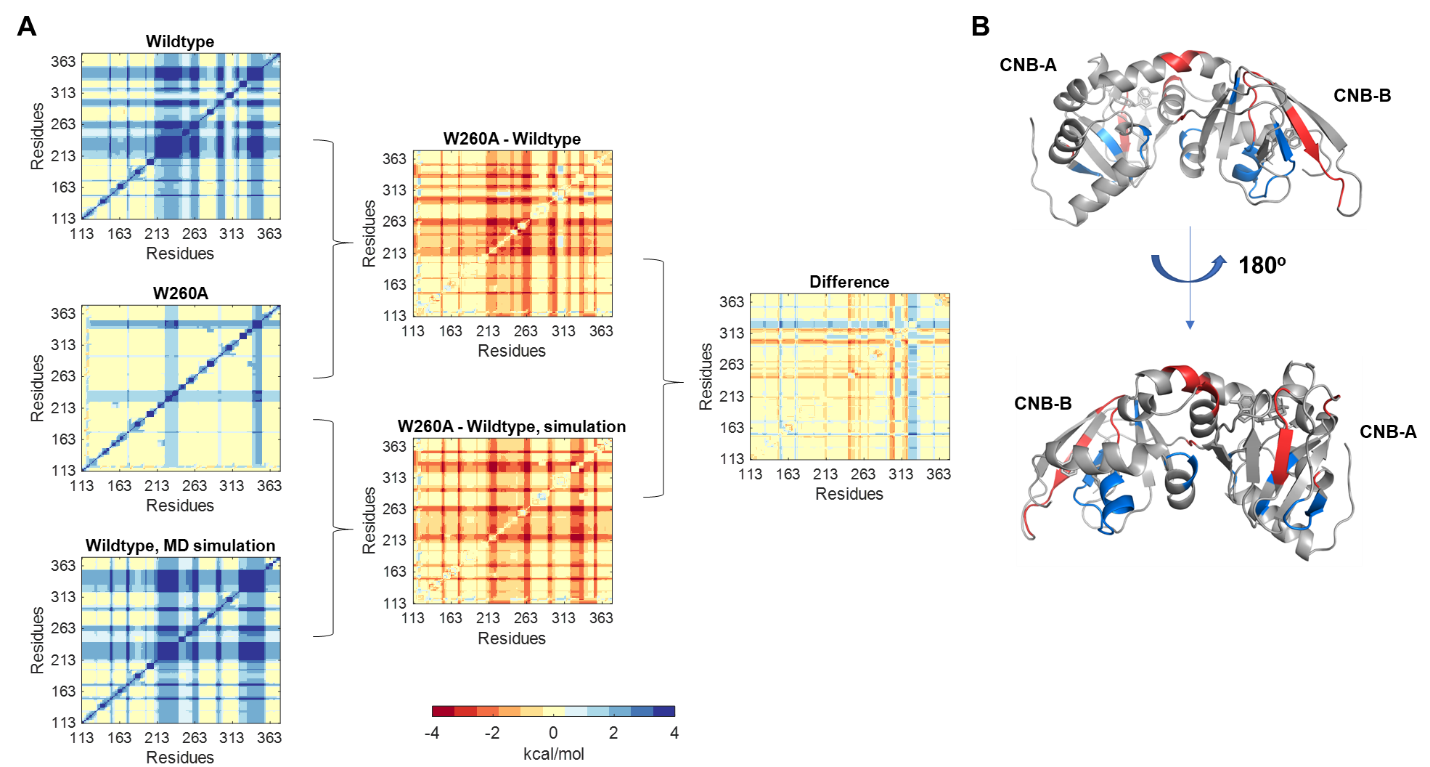


Fig. S3. Comparison between crystal structure and molecular dynamics simulation. A. cAMP-bound R-subunit COREX results from the wildtype crystal structure (PDB:1RGS), molecular dynamics (MD) simulation of W260A, and MD simulation of 1RGS were generated. To account for the effect of the MD simulation in the comparison between wildtype and mutant R subunits, the differences of W260A minus wildtype and W260A minus wildtype, simulation were calculated. B. The differences due to the MD simulations were shown on the cAMP-bound R-subunit structure. Blue depicts higher coupling in the simulation, whereas red depicts lower coupling in the simulation The difference distributions were shown throughout the structure.


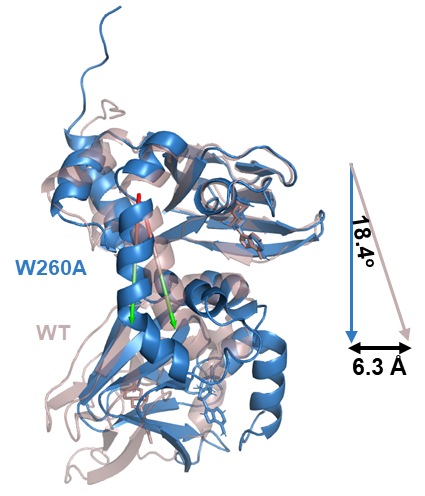


Fig. S4. Alignment of wildtype and W260A R-subunits. The crystal structure 1RGS and the MD simulation W260A R-subunit are aligned at the CNB-A domain (residues 113-242). The rotational angle and the displacement of the CNB-B domain is determined using PyMOL script angle_between_domains written by Thomas Holder. Script can be obtained on <https://github.com/speleo3/pymol-psico/blob/master/psico/orientation.py>.

Table S1. Kinetic parameters of wildtype (WT) and W260A mutant R-subunit in apo, cAMP-bound, and C-subunit-bound states.

Bracket indicates the percentage of CNB-B unfolding with the ΔLc fit.

| **Apo** | | **Unfolding Force (pN)** | **τ_0_ (s)** | **Δx^ⱡ^ (nm)** | **ΔLc (nm)** |
| --- | --- | --- | --- | --- | --- |
| WT | CNB-A  (n = 455) | 10.5 ± 1.0 | 5.8 ± 2.4 × 10^4^ | 4.8 ± 0.2 | 44.2 ± 0.1 |
|  | CNB-B  (n = 455) | 9.2 ± 0.9 | 9.1 ± 3.4 × 10^3^ | 4.8 ± 0.2 | 50.3 ± 0.1 |
| W260A | CNB-A  (n = 820) | 11.3 ± 1.4 | 3.6 ± 2.2 × 10^4^ | 4.1 ± 0.2 | 46.6 ± 3.3 |
|  | CNB-B  (n = 820) | 8.8 ± 1.5 | 3.8 ± 1.0 × 10^2^ | 3.2 ± 0.1 | 50.3 ± 3.8 |
|  | |  |  |  |  |
| **cAMP-bound** | | **Unfolding Force (pN)** | **τ_0_ (s)** | **Δx^ⱡ^ (nm)** | **ΔLc (nm)** |
| WT | CNB-A  (n = 728) | 17.2 ± 1.6 | 1.6 ± 0.6 × 10^5^ | 3.0 ± 0.1 | 30.4 ± 4.7 |
|  | CNB-B  (n = 669) | 14.8 ± 1.5 | 5.3 ± 1.2 × 10^3^ | 2.7 ± 0.1 | 49.4 ± 4.0 |
| W260A | CNB-A  (n = 2756) | 17.7 ± 1.5 | 5.3 ± 1.1 × 10^4^ | 2.7 ± 0.05 | 31.4 ± 3.2 |
|  | CNB-B  (n = 2755) | 13.1 ± 1.0 | 8.8 ± 2.8 × 10^5^ | 4.7 ± 0.1 | 39.5 ± 3.3 [80%]  53.6 ± 2.5 [20%] |
|  | |  |  |  |  |
| **C-subunit-bound** | | **Unfolding Force (pN)** | **τ_0_ (s)** | **Δx^ⱡ^ (nm)** | **ΔLc (nm)** |
| WT | CNB-A  (n = 1140) | 16.8 ± 2.7 | 4.1 ± 0.8 × 10^2^ | 1.6 ± 0.1 | 47.5 ± 2.7 |
|  | CNB-B  (n = 1140) | 14.4 ± 2.8 | 1.7 ± 0.2 × 10^2^ | 1.6 ± 0.05 | 51.6 ± 6.0 |
| W260A | CNB-A  (n = 499) | 18.1 ± 3.0 | 3.6 ± 1.0 × 10^2^ | 1.4 ± 0.1 | 46.6 ± 3.3 |
|  | CNB-B  (n = 499) | 14.4 ± 2.5 | 7.4 ± 2.9 × 10^2^ | 2.0 ± 0.1 | 50.3 ± 3.8 |

Table S2. Kinetic parameters of wildtype (WT) and W260A truncated CNB-B domain in apo and cAMP-bound state.

Bracket indicates the percentage of CNB-B unfolding in each conformation.

^1^Kinetic parameters obtained from Hao *et al*. (*11*)

| **Apo** | **Unfolding Force (pN)** | **τ_0_ (s)** | **Δx^ⱡ^ (nm)** | **ΔLc (nm)** |
| --- | --- | --- | --- | --- |
| WT^1^  (n = 744) | 7.3 ± 1.2 | 4.0 ± 1.1 × 10^2^ | 4.1 ± 0.2 | 50.3 ± 2.7 |
| W260A  (n = 2930) | 7.3 ± 1.1 | 1.6 ± 0.2× 10^3^ | 4.9 ± 0.1 | 50.1 ± 4.2 |
|  |  |  |  |  |
| **cAMP-bound** | **Unfolding Force (pN)** | **τ_0_ (s)** | **Δx^ⱡ^ (nm)** | **ΔLc (nm)** |
| WT  (n = 506) | 12.6 ± 0.9 | 1.3 ± 0.8 × 10^5^ | 4.3 ± 0.2 | 40.7 ± 2.7 [48%]  47.0 ± 2.8 [51%] |
| W260A  (n = 4646) | 12.6 ± 0.9 | 3.7 ± 0.8 × 10^5^ | 4.6 ± 0.1 | 40.4 ± 2.1 [66%]  49.9 ± 3.0 [34%] |

Table S3. Percentage of observing unfolding of one step or two steps in CNB-B in R-subunit and truncated domain by varying refolding times.

Notation reads [percentage of unfolding in two steps]/[percentage of unfolding in one step].

WT = wildtype

n.d. = not determined

| **Refolding time (s)** | **WT truncated CNB-B** | **WT R-subunit** | **W260A truncated CNB-B** | **W260A R-subunit** |
| --- | --- | --- | --- | --- |
| 1 | 59.0 ± 0.2 / 41.0 ± 0.2 | 5.0 ± 6.6 / 95.0 ± 6.6 | 66.7 ± 0.2 / 33.3 ± 0.2 | n.d. |
| 5 | 45.8 ± 0.2 / 54.2 ± 0.2 | 10.9 ± 0.1 / 89.1 ± 0.1 | 53.9 ± 0.1 / 46.1 ± 0.1 | n.d. |
| 10 | 53.2 ± 0.1 / 46.8 ± 0.1 | 7.6 ± 0.1 / 92.4 ± 0.1 | 66.4 ± 0.3 / 33.6 ± 0.3 | 80.5 ± 0.1 / 19.5 ± 0.1 |

Table S4. Kinetic parameters of W260A R-subunit in apo, intermediate (A-bound and B-bound) and fully-bound states.

Bracket indicates the percentage of CNB-B unfolding with the ΔLc fit.

| **CNB-A** | **Unfolding Force (pN)** | **τ_0_ (s)** | **Δx^ⱡ^ (nm)** | **ΔLc (nm)** |
| --- | --- | --- | --- | --- |
| Apo  (n = 820) | 11.33 ± 1.50 | 3.2 ± 1.4 × 10^4^ | 4.1 ± 0.2 | 46.6 ± 3.3 |
| A-bound  (n = 190) | 16.88 ± 2.18 | 2.5 ± 1.7 × 10^3^ | 2.0 ± 0.2 | 30.9 ± 2.2 |
| B-bound  (n = 546) | 11.72 ± 2.17 | 2.1 ± 0.8 × 10^6^ | 4.9 ± 0.2 | 44.9 ± 4.4 |
| AB-bound  (n = 2756) | 17.71 ± 1.54 | 5.5 ± 1.0 × 10^4^ | 2.7 ± 0.1 | 30.8 ± 2.1 |
|  |  |  |  |  |
| **CNB-B** | **Unfolding Force (pN)** | **τ_0_ (s)** | **Δx^ⱡ^ (nm)** | **ΔLc (nm)** |
| Apo  (n = 820) | 8.79 ± 1.37 | 4.0 ± 0.1 × 10^2^ | 3.3 ± 0.1 | 50.3 ± 3.8 |
| A-bound  (n = 190) | 8.00 ± 1.70 | 1.0 ± 0.1 × 10^2^ | 2.8 ± 0.1 | 49.7 ± 4.7 |
| B-bound  (n = 546) | 13.39 ± 0.83 | 1.1 ± 0.4 × 10^4^ | 3.5 ± 0.1 | 40.1 ± 3.2 [60%]  51.5 ± 2.6 [40%] |
| AB-bound  (n = 2755) | 13.11 ± 1.02 | 8.2 ± 2.7 × 10^5^ | 4.7 ± 0.1 | 39.5 ± 3.3 [80%]  53.6 ± 2.5 [20%] |

Table S5. cAMP binding affinity of wildtype (WT) and W260A PKA R-subunit and truncated CNB domains in different cAMP occupancy states.

^1^Parameters obtained from Hao *et al*. (*11*)

| **cAMP binding step** | **WT^1^ (10^7^ M^-1^)** | **W260A (10^7^ M^-1^)** |
| --- | --- | --- |
| A_0_B_0_ $\underset{\to}{k_{1}}$ A_1_B_0_ | 5.7 ± 0.5 | 1.9 ± 0.1 |
| A_0_B_0_ $\underset{\to}{k_{2}}$ A_0_B_1_ | 10 ± 1.0 | 4.9 ± 0.4 |
| A_1_B_0_ $\underset{\to}{k_{3}}$ A_1_B_1_ | 15 ± 2.0 | 1.9 ± 0.1 |
| A_0_B_1_ $\underset{\to}{k_{4}}$ A_1_B_1_ | 8.7 ± 1.2 | 4.9 ± 0.4 |
| Cooperativity | 1.4 | 1.0 |
|  |  |  |
| **K_A_** | **WT^1^ (10^7^ M^-1^)** | **W260A (10^7^ M^-1^)** |
| Truncated CNB-A domain | 1.2 ± 0.3 | -- |
| Truncated CNB-B domain | 3.7 ± 0.3 | 5.9 ± 0.6 |
